# Tumor microenvironment-responsive hyperbranched polymers for controlled drug delivery

**DOI:** 10.1016/j.jpha.2024.101003

**Published:** 2024-05-22

**Authors:** Yuqiong Guo, Xinni He, Gareth R. Williams, Yue Zhou, Xinying Liao, Ziyi Xiao, Cuiyun Yu, Yang Liu

**Affiliations:** a Hunan Provincial Key Laboratory of Tumor Microenvironment Responsive Drug Research, Hunan Province Cooperative Innovation Center for Molecular Target New Drug Study, School of Pharmaceutical Science, Hengyang Medical School, University of South China, Hengyang, Hunan, 421001, China; b UCL School of Pharmacy, University College London, London, WC1N1AX, UK

**Keywords:** Drug delivery, Hyperbranched polymer, Stimuli responsive, Tumor microenvironment

## Abstract

Hyperbranched polymers (HBPs) have drawn great interest in the biomedical field on account of their special morphology, low viscosity, self-regulation, and facile preparation methods. Moreover, their large intramolecular cavities, high biocompatibility, biodegradability, and targeting properties render them very suitable for anti-tumor drug delivery. Recently, exploiting the specific characteristics of the tumor microenvironment, a range of multifunctional HBPs responsive to the tumor microenvironment have emerged. By further introducing various types of drugs through physical embedding or chemical coupling, the resulting HBPs based delivery systems have played a crucial part in improving drug stability, increasing effective drug concentration, decreasing drug toxicity and side effects, and enhancing anti-tumor effect. Here, based on different types of tumor microenvironment stimulation signals such as pH, redox, temperature, etc., we systematically review the preparation and response mechanism of HBPs, summarize the latest advances in drug delivery applications, and analyze the challenges and future research directions for such nanomaterials in biomedical clinical applications.

## Highlights

• HBPs have attracted much attention in controlled drug delivery.
• Various tumor microenvironment-responsive HBPs in tumor therapy were summarized.
• The preparation basis and mechanism of responsive HBPs were introduced.
• Representative HBPs for drug delivery in tumors were further expanded.

## Graphical abstract

**Figure:**
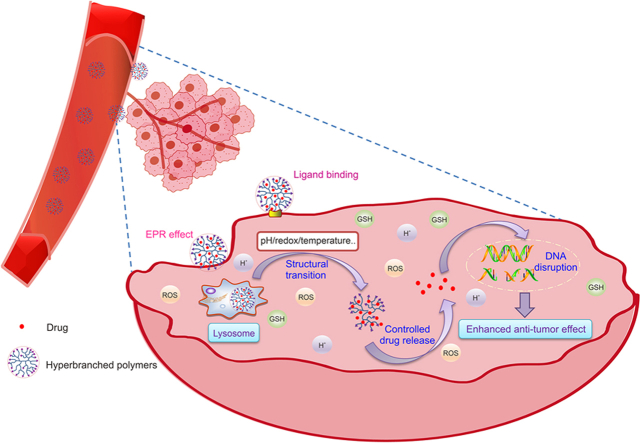

## 1 Introduction

Tumor is one of the most prevalent diseases that severely endanger life and health. Therefore, searching for an efficient and low-toxicity tumor therapeutic method has become a key research objective of scholars internationally. In addition to surgical resection, drug therapy is one of the commonly used clinical treatments. However, the commonly used small molecule drugs often have problems of low solubility, poor selectivity, significant side effects, and susceptibility to multidrug resistance. In particularly, their low molecular weight characteristics make them extremely easy to be cleared by the blood and kidneys, making it difficult for them to accumulate efficiently in tumor sites. The construction and preparation of targeted drug delivery systems has thus been widely explored [[1], [2], [3]]. Such systems can not only effectively enhance the drug loading, increase the *in vivo* circulation time, and boost the bioavailability, but also aid drugs to accumulate in tumor tissues through flexible application of passive or active targeting mechanisms, thereby improving the efficacy of drugs and decreasing the toxicity on healthy cells or tissues.

With the efforts of researchers from various disciplines, numerous delivery systems such as macromolecular prodrugs [[4], [5], [6]], nanoparticles [[7], [8], [9]], micelles [[10], [11], [12]], liposomes [[13], [14], [15]], nanogels [[16], [17], [18]] and hydrogels [[19], [20], [21], [22]] have been investigated and developed. Among them, hyperbranched polymers (HBPs) have attracted great interest among researchers [[23], [24], [25]]. The terminology and definition of HBPs was introduced by Kim and Webster [26] for the first time in 1990, and defines them as a specific kind of dendritic macromolecule with stochastic branch-on-branch topology. The highly branched structure of HBPs makes them have vast intramolecular cavities, and the size of their intramolecular cavities can be adjusted by control of their molecular weight, branching degree and intramolecular cyclization, so that payloads of various types of drugs can be incorporated through physical embedding. In addition, HBPs have richer reactive groups in linear, branching, and terminal sites compared to linear polymers, which are susceptible to chemically or targeted modification. Therefore, drug molecules can also be efficiently loaded into HBPs through strategies such as chemical bonding or intermolecular forces. The sizes of HBPs are generally in the range of several nanometers to dozens of nanometers, and they can be further assembled into vesicles, micelles, and other supramolecular systems [[27], [28], [29]]. Thus, they have passive targeting characteristics, which can extend the time of circulation *in vivo* and increase the retention of drugs in tumors. In contrast to linear polymers, HBPs have higher solubility and lower viscosity, while compared with dendrimers and other dendritic macromolecules, HBPs can generally be prepared by a one-step reaction, which is simple and less costly [[30], [31], [32]]. Moreover, HBPs have better structural freedom and are easier to chemically modify, so HBPs show advantages and application prospects in drug delivery [[33], [34], [35]].

Compared with healthy tissues, tumor tissue has a microenvironment that can accelerate tumor formation and proliferation, cause neovascularization, suppress the response, and lead to drug tolerance. This is characterized by a lower pH value, higher temperature, greater concentration of reducing substances, and other physiological characteristics [[36], [37], [38]]. Thus, it is of great significance to make full use of these features of the tumor microenvironment to construct anti-tumor drug delivery systems which respond specifically to the abnormal characteristics of the tumor tissue [[39], [40], [41]]. Due to the ease of structural modification and functional tunability at branching, linear, and terminal units, it is very easy to introduce stimuli-responsive groups or units into HBPs by means of monomer design and group modification, so as to make the HBPs responsive to the tumor microenvironment and realize controlled drug delivery [42]. Consequently, the construction of HBPs based drug delivery systems with tumor microenvironmental responsiveness is highly attractive to researchers.

**Fig. 1:**
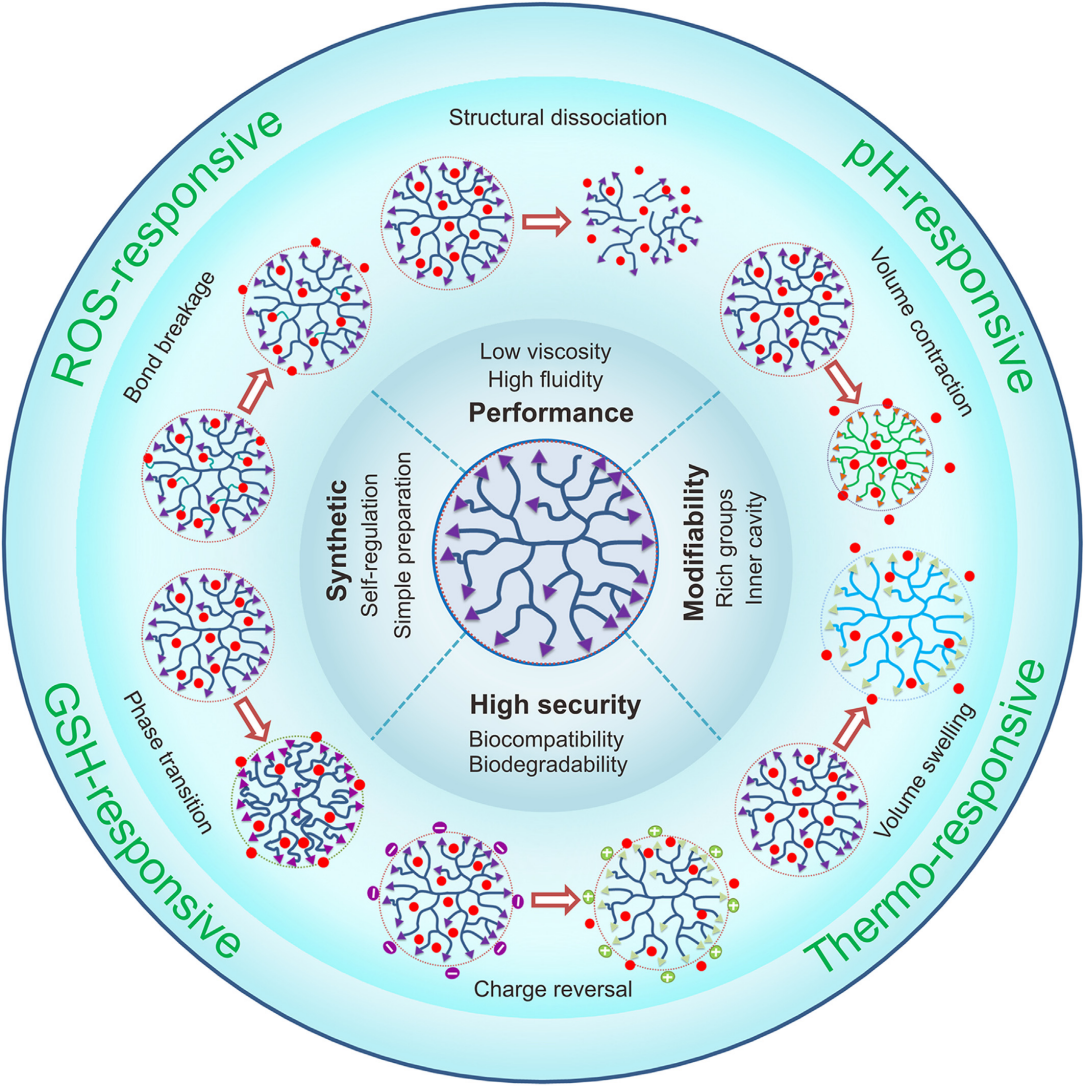
Scheme of tumor microenvironment-responsive hyperbranched polymers (HBPs) for controlled drug delivery. ROS: reactive oxygen species; GSH: glutathione.

Therefore, we conducted a literature search through Web of Science to get an overview of tumor microenvironment-responsive HBPs for controlled drug delivery. Using HBPs, delivery, responsive, etc. as relevant keywords, we searched the literature published in the past 10 years, and through further screening and related literature searches we finally obtained 130 relevant articles approximately. Based on this, we systematically reviewed the synthesis method and stimulus response mechanism of responsive HBPs responding to different types of stimuluses, such as pH, redox, temperature, and multiple-stimuli (Table 1). This review summarizes the recent research progress in developing tumor microenvironment-responsive HBPs based drug delivery systems (Fig. 1), and discusses the development prospects and likely hurdles to further progress the application of stimuli-responsive HBPs in tumor therapy.

**Table 1:** Characteristics of hyperbranched polymers (HBPs) based drug delivery systems with different tumor microenvironment-responsiveness.

Responsiveness	Classification	Advantage	Disadvantage
pH-responsive	Acid-labile chemical bond and ionizable group	High response sensitivity, wide pH regulation range, and high drug release controllability	Small pH gradient variation and complex *in vivo* pH microenvironment
Redox-responsive	ROS-responsive and GSH-responsive	High stability, high response sensitivity, and sharp specificity	Possible over-generated ROS, different ROS levels among different tumors, and preparation is a little difficult
Thermo-responsive	Thermosensitive monomer and thermosensitive structure	Easily adjustable thermo-sensitivity, wide temperature regulation range, and facile preparation	Need to combine with another stimulus, uncertain stability, and limited clinical application
Multiple-responsive	pH/redox dual-responsive, pH/thermo dual-responsive, and multi-stimuli-responsive	Combination of endogenous or exogenous stimulus-responsive and more precise regulation of drug release	Complex synthesis steps and difficulty in scaling and commercialization

ROS: reactive oxygen species; GSH: glutathione.

## 2 pH-responsive HBPs

The respiration mode of tumor cells is dominated by aerobic glycolysis, so the microenvironment of tumor is weakly acidic with a pH around 5.0–6.0, and the pH in primary and secondary lysosomes can even be as low as 4.0–5.0, both well below the natural physiological environment (pH 7.4). Therefore, when a drug delivery system containing a pH-sensitive structure or moiety reaches the tumor site through blood circulation, it will selectively undergo structural changes due to the difference in pH, thereby releasing the loaded drug and achieving selective suppression of the growth of tumor cells. In addition, if the delivery system can be further transferred into the cell via endocytosis, it will undergo further structural changes or even degradation in organelles such as lysosomes, leading to faster drug release. Thus, pH-responsive drug delivery systems have drawn considerable interest in tumor therapy [[43], [44], [45]]. Due to the favorable structural tunability of HBPs, it is very easy to introduce pH-sensitive groups or units into them through various measures, mainly including acid-labile chemical bonds and ionizable groups, so as to construct pH-responsive drug delivery systems and exert efficient anti-tumor effects.

### 2.1 pH-responsive HBPs with acid-labile chemical bonds

Polymers containing dynamic chemical bonds are an important branch of smart nanomaterials [46,47]. The presence of dynamic chemical bonds not only improves the stability of polymers, but also makes them structurally reversible. When exposed to various stimuli factors, they can reorganize or optimize their network structure through the breaking and re-forming of dynamic covalent bonds to adapt to the external environment, and thus they have gained attention in biomedical fields, such as drug release, tissue repair, and so on [48]. Chemical bonds such as Schiff base bonds, acylhydrazone bonds, and acetal bonds are pH-sensitive and show reversible cleavage under specific acidity conditions. Therefore, nano-drug delivery systems constructed by introducing these bonds into HBPs can undergo structural changes in the tumor microenvironment, thus achieving acid-triggered drug release. Depending on the structure or the location of the pH-responsive covalent bonds, we can classify such pH-responsive HBPs into two categories: one is pH-responsive HBPs with responsive skeletons, in which the pH-responsive covalent bonds are located on the skeleton of HBPs. The other is pH-responsive HBPs with responsive terminal groups, which uses the terminal groups on HBPs to form acid-labile chemical bonds through surface modification.

**Fig. 2:**
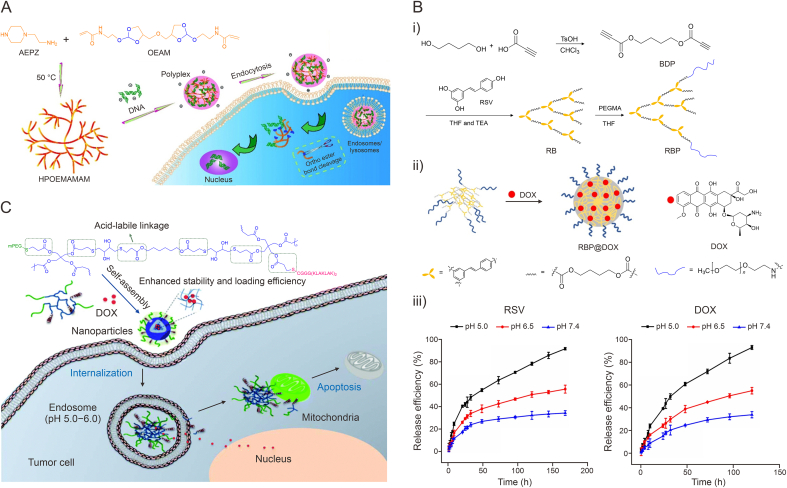
pH-responsive hyperbranched polymers (HBPs) with responsive skeletons obtained from the reactions between monomers without pH-responsiveness. (A) Schematic illustration of the synthesis of poly(ortho ester amido amine) HBPs containing pH-sensitive ortho ester bonds (HPOEAMAM) and gene delivery *in vitro* [50]. (B) Schematic illustration of the synthesis of pH-responsive degradable hyperbranched polymer (RBP) and the controlled release curves of doxorubicin (DOX) and resveratrol (RSV) [52]. (C) Schematic illustration of the (KLAKLAK)_2_ (KLAK) peptide-conjugated pH-responsive hyperbranched poly(β-thioester)s (PPHD-PK) for combined therapy [53]. AEPZ: *N*-aminoethylpiperazine; OEAM: *N*,*N*ʹ-((((oxybis(methylene))*bis*(1,3-dioxolane-4,2-diyl))*bis*(oxy))*bis*(ethane-2,1-diyl))bis(2-methylacrylamide); TsOH: p-toluene sulfonic acid; BDP: *n*-butyl dipropiolate ; THF: tetrahydrofuran; TEA: triethylamine; PEGMA: amine-terminated methoxy poly(ethylene glycol) amine; RB: hyperbranched DOX polyprodrug; mPEG: methoxy poly(ethylene glycol). Reprinted from Refs. [50,52,53] with permission.

pH-responsive HBPs with responsive skeletons are generally obtained by introducing pH-sensitive chemical bonds into the polymerization reaction monomers, or by utilizing reactions between several monomers. Thus, they can be subdivided into two groups according to the nature of the monomers. One is where the polymerized monomers are not pH-responsive by themselves, but can form acid-labile chemical bonds after a series of reactions, which endow HBPs with pH-responsiveness. For instance, hyperbranched polyester derivatives obtained by condensation reactions between monomers containing hydroxyl groups may be degraded under acidic conditions due to accelerated breakage of ester bonds on the backbone, and the products are also highly biocompatible, making them highly suitable as pH-sensitive anti-tumor drug delivery carriers. Öztürk et al. [49] developed a type of polyethylene glycol (PEG) linked, dl-lysine (Lys) and folic acid (FA) modified, 3rd generation HBPs (HBP-PEG-Lys-FA) for tumor treatment. 5-fluorouracil (5-FU) was able to be loaded with a loading efficiency of 23.18%. The existence of ester groups in the structure endowed the carrier with good pH-responsiveness, and after 24 h in buffer solution at pH 5.5, 68% of the 5-FU was released from the polymer versus only 26% at pH 7.4. Due to the existence of FA, the nano-carrier could be efficient in delivering drugs to the cytoplasm of cancer cells, and comprised a safe and effective delivery system. Li et al. prepared a type of novel poly(ortho ester amido amine) (HPOEAMAM) HBPs with acid-labile ortho esters bonds via Michael addition polymerization and explored these for gene delivery (Fig. 2A) [50]. HPOEAMAM was capable of forming a stable polyplex with plasmid DNA via electrostatic force under neutral conditions, and releasing DNA due to hydrolysis of ortho ester bonds under acidic conditions. Both in two-dimensional (2D) cultured SH-SY5Y cells and 3D multicellular tumor spheroids, it showed significantly improved gene transfection efficiency.

Click reactions such as alkyne-thiol, alkyne-azide, and alkene-thiol are also commonly used to prepare HBPs [51]. Among them, HBPs constructed through click coupling between acyl alkynyl and phenolic or amines comprise a class of pH-responsive drug carriers because the vinyl ether or vinyl amine in their structures can be destroyed at low pH conditions. For instance, Zhang et al. constructed a degradable pH-responsive hyperbranched polymer (RB) through alkyne-phenol click coupling between resveratrol (RSV) and *n*-butyl dipropiolate (BDP) (Fig. 2B) [52]. Furthermore, through the click reaction with amino terminally modified PEG (mPEG-NH_2_), PEGylated RB (RBP) was synthesized, which improved the water dispersity of the nano-carrier and effectively prolonged its blood circulation time. The RBP had a high drug loading rate of doxorubicin (DOX) at neutral pH (58.6%), but under the low pH of the tumor microenvironment, DOX and RSV could be released more quickly, due to dissociation of the vinyl ether bonds, thus made them selectively act on the tumor site. In addition, RSV had antioxidant activity, which could protect healthy tissues from the side effects of DOX; therefore, RSV could produce synergistic effects with DOX to further enhance the therapeutic effects.

β-thiopropionate is also an attractive acid-labile chemical bond that is often applied in the preparation of pH-responsive nano-carriers. Cheng et al. prepared a proapoptosis (KLAKLAK)_2_ (KLAK) peptide conjugated pH-responsive hyperbranched poly(β-thioester) via a one pot reaction, which could assemble to form nanoparticles for DOX loading (Fig. 2C) [53]. The obtained nanoparticles (PPHD-PK) were well colloidal stable with 8.2% drug loading capacity (DLC) and 80.1% drug loading efficiency (DLE). At pH 7.4, less than 35% of DOX was gradually released within 48 h. While under acidic condition, the release was expedited by thiopropionate disruption, and cumulative release rate could reach 80%. The particles were endocytosed by tumor cells and released the drug cargo in the acidic lysosomal environment, thus playing an anti-tumor role. The KLAK peptide could also exert a synergistic anti-tumor effect to expand the therapeutic effect.

Liang et al. [54] first synthesized a type of poly(amino ester) based HBPs (HBPAE-OH) via the Michael addition polymerization, with internal hydroxyl and terminal acrylate groups in their structures. The surface of HBPAE-OH was then coated with PEG chains, and DOX were covalently loaded into the core via acid-labile hydrazide bonds. This polymer could form a spherical unimolecular micelle whose size was around 14.2 nm. The results demonstrated that due to the fission of hydrazide bonds under acidic conditions, about 91.8% of the DOX was rapidly released within 12 h at pH 6.0, while the release in physiological conditions was very slow and reached a plateau at 6 h with a cumulative release rate of less than 20%. Duan et al. [55] synthesized an AB_2_ monomer, 2-hydroxyethyl-4-formylbenzoate, through the reaction between 4-carboxybenzaldehyde and 2-bromoethanol, and obtained a hyperbranched polyacetal through the polycondenzation of this monomer. Further, this polyacetal was coupled with mPEG-NH_2_ and DOX on their surfaces via Schiff base bonds, resulting in an amphiphilic pH-sensitive polymer-drug conjugate (DOX-hyperbranched polyamide (HBPA)-PEG). This was able to self-assemble into a stable micelle under physiological pH conditions, while dissociating under acidic conditions due to the existence of acetal and Schiff base bonds. The cumulative release rate of DOX after 24 h was 48.56% at pH 6.0 and 14.51% at pH 7.4. The micelles could be fully endocytosed by A549 cells within 1 h, thus exerting effective anti-tumor effects.

**Fig. 3:**
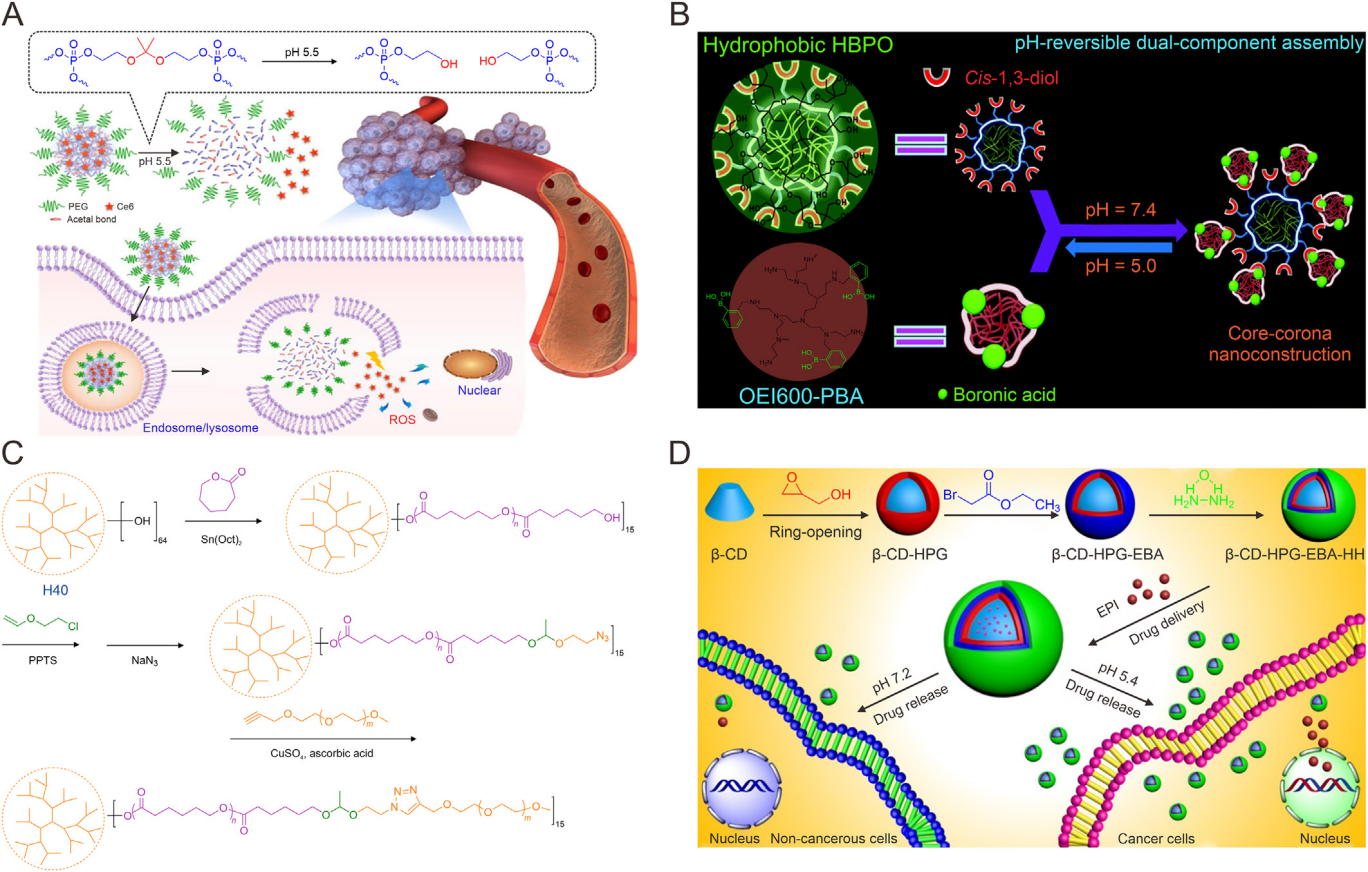
pH-responsive hyperbranched polymers (HBPs) with responsive skeletons obtained from the polymerization reaction of pH-responsive monomers. (A) Schematic illustration of the hyperbranched polyphosphoric ester (*S*-hbPPE) linked with pH-sensitive acetal bonds for the delivery of chlorin e6 (Ce6) [56]. (B) Schematic illustration of the acid-responsive corona-core carrier from hyperbranched oligoethylenimine (OEI) modified with phenylboronic acid (PBA) [63]. (C) Schematic illustration of the synthesis pathways of acetal linked Boltorn H40 (H40)-star-poly(ε-caprolactone) (PCL)-a-poly(ethylene glycol) (PEG) polymers [65]. (D) Schematic illustration of the preparation of hyperbranched β-cyclodextrin (β-CD)-hyperbranched polyglycerol (HPG)-ethyl bromoacetate (EBA)-hydrazine hydrate (HH) for drug delivery [66]. ROS: reactive oxygen species; HBPO: HPG with a substantial 1,3-diol structures; Sn(Oct)_2_: tin(II) 2-ethylhexanoate; PPTS: pyridinium *p*-toluenesulfonate; EPI: epirubicin. Reprinted from Refs. [56,63,65,66] with permission.

Other pH-responsive HBPs with responsive skeletons are generally obtained by polymerization reaction using monomers with pH-responsiveness. Although the monomer structures in the final polymer are changed, the stimulus-responsive groups are retained. Among these, molecules containing acetal bonds are one of the common pH-responsive monomers. For instance, Li et al. synthesized an hyperbranched polyphosphoric ester (*S*-hbPPE) via a condensation reaction between 2,2′-(propane-2,2-diylbis(oxy)) diethanol containing an acetal bond, PEG, and phosphoryl chloride, for encapsulation of the chlorin e6 (Ce6) photosensitizer (Fig. 3A) [56]. This formulation could achieve extensive release of Ce6 at the tumor site. The size of *S*-hbPPE/Ce6 decreased from 112 ± 5 nm to 42 ± 4 nm after 12 h at pH 5.5. Therefore, it could be assumed that the acetal bonds in this novel pH-sensitive system can rapidly break in acidic endosomes and lysosomes, leading to structure dissociation, thus facilitating the release of Ce6. The results suggested that 27% of Ce6 was released within 20 h under physiological conditions, while the release reached 50% at pH 5.5. When treated with this nanoparticle, the intracellular Ce6 content of BxPC-3 cells was more than twice as much as those treated with free Ce6, and more reactive oxygen species (ROS) and more significant inhibition of cell growth could be found. Tumor animal experiments also showed that *S*-hbPPE could extend the blood circulation time of Ce6 and exhibit photodynamic therapy (PDT) based anti-tumor effects.

However, if the drug leaks before reaching the intended site, it will cause certain side effects, so to improve the carrier stability under physiological conditions and reduce the possibility of its non-essential release, we can introduce divinyl-like groups into the pH-responsive monomer structure to make hyperbranched nuclei for further crosslinking to improve the structure stability. For example, Cao et al. [57] synthesized an amphiphilic branched polymer with hydrophobic divinyl units connected by pH-responsive acetal groups and hydrophilic PEG fragment. The method introduced abundant vinyl groups that could be further modified and prevent the premature drug leakage by further crosslinking. The authors investigated the drug release behavior in the micelles using DOX as the guest drug and found that the micelles showed less release at both pH 5.4 and 7.4 compared to nuclear non-crosslinked micelles. 3-(4,5-Dimethylthiazol-2-yl)-2,5-diphenyltetrazolium bromide (MTT) assay results showed no significant cytotoxicity of the empty micelles, whereas the micelles loaded with DOX showed significant anti-tumor effects. In other work, Li and Liu [58] firstly synthesized a DOX based prodrug monomer (MH-DOX) with pH-sensitive bond through the conjugation of DOX with 2-aminoethyl methacrylamide (AEMA) via a hydrazone bond. After further polymerization reaction of MH-DOX with PEG methacrylate (PEGMA), a HBP prodrug was obtained, which could form an unimolecular micelle with a size of approximately 130 nm. This micelle could maintain excellent stability, with only 3.9% premature drug leakage after 58.5 h at pH 7.4, thus being promising to reduce toxicity for normal cells or tissues *in vivo*. Cumulative DOX release at pH 5.0 could reach 80% within 24 h. This micelle was able to be endocytosed by HepG2 cells and induced inhibition of cell proliferation. In addition, the micelle was also able to produce a stronger inhibitory effect on DOX-resistant breast cancer cells than free DOX, showing great potential in inhibiting drug resistance of cells.

pH-responsive HBPs with responsive terminal groups generally take advantage of the presence of terminal surface reactive groups to covalently bond other small molecules or oligomers, or to graft stimuli-responsive polymers, so as to confer them pH-responsiveness. For instance, Yu et al. [59] prepared a biocompatible hyperbranched polyhydrazide (HPAH) with pH-responsive by the introduction of acylhydrazone bonds using the condensation reaction of diketones and trihydrazones, followed by the connection of hydrophilic PEG to the hyperbranched core. In paclitaxel (PTX) release experiments, it was found that after endocytosis, the acylhydrazone bonds of HPAH was broken under acidic conditions (pH 5–6), and drug release was increased. When studied with tumor-bearing mice, HPAH was found to have better anti-tumor effect and biodistribution in mice over a period of three weeks with lower toxicity compared to free PTX. In addition, Yan et al. [60] obtained a methoxy PEG (MPEG)-b-polyethyleneimine (PEI)-poly(Nε-Cbz-l-lysine) (PBLL) based pH-responsive micelle carrier (MPEG-PEI-PBLL) by using Schiff base bonds as the linker. The system had a large loading rate of DOX (25.8%), and the cumulative release of the drug was more favorable at low pH due to the presence of Schiff base linkages. For example, after release for 72 h, the cumulative release (pH 5.0) reached 78.4%, whereas it was only 19.3% at pH 7.4. Cellular experiments showed that it still had cell growth-inhibiting activities, and animal experiments suggested that the formulation could accumulate in tumors and produce reduced side effects such as myocardial toxicity, thus making it a potential delivery vehicle for anti-tumor drugs. Chen and Wang [61] directly connected PEG chains through hydrazone bonds on pH-responsive hyperbranched polyacetal, and obtained a hyperbranched polymeric micelle (HBPAs-hydrazone-PEG) containing two pH-responsive groups, which also showed superior pH responsiveness to DOX release and has great potential for application.

Phenylboronic acid (PBA) ester bonds are generated via coupled reaction of PBA with substances having the structure of vicinal diols (1,2-diol or 1,3-diol) at higher pH, which are prone to fracture under acidic conditions, and thus also have good pH-responsiveness [62]. Jia et al. coupled hyperbranched oligoethylenimine modified with PBA (OEI-PBA) on the surface of hyperbranched polyglycerol (PG) with a substantial 1,3-diol structures (HBPO) via borate ester bond to construct an acid-responsive corona-core carrier for drug/gene co-delivery (Fig. 3B) [63]. Compared with OEI-PBA, the carrier exhibited superior ability to snatch DNA due to the special corona-core structure, with about 10 times higher cell absorption efficiency and 100 times higher transfection activity. The carrier was also capable of further loading DOX, and the cumulative DOX release rate under acidic condition was much higher than that at physiological pH. Using HeLa cells as the model, the carrier co-loaded with DOX and *p53* gene was found to produce stronger inhibition of cell growth. The authors further employed this carrier to delivery *Beclin1* small interfering RNA (siRNA) and DOX to suppress cell survival after chemotherapy resulted from drug-induced autophagy, further expanding the application scope of this type of carrier in tumor therapy [64].

However, the supramolecular interactions between polymer chains are weak, and the multimolecular micelles prepared by HBPs are prone to disintegrate after significant dilution of blood, resulting in drug leakage and other problems during blood circulation. To overcome this challenge, Zhang et al. prepared star copolymer based monomolecular micelles (Boltorn H40 (H40)-star-poly(ε-caprolactone) (PCL)-a-PEG), which were formed using acetal groups as linkers (Fig. 3C) [65]. The acid cleavage of the acetal groups could give the system pH-responsiveness. The results suggested that the release of DOX was less than 20% at neutral pH, but more than 70% at pH 5, suggesting that the monomolecular micelles are more stable in neutral aqueous solution, while the decomposition occurred in acid. Experiments also suggested that they were remarkably internalized into the HeLa cells to exert excellent anti-tumor effect.

In addition, pH-responsive HBPs obtained by direct coupling of drugs to the end groups of HBPs via acid-sensitive dynamic bonds also have gained prominence in the last few years. For instance, Wan et al. obtained a hyperbranched β-cyclodextrin (β-CD) modified polymer (β-CD-hyperbranched polyglycerol (HPG)-ethyl bromoacetate (EBA)-hydrazine hydrate (HH)) with hydrazide terminus (Fig. 3D) [66]. Since the hydrazide groups could react with the anti-tumor drug epirubicin (EPI) to form hydrazone bonds, the authors constructed a HBP prodrug with pH-responsive. The cumulative release of EPI from this prodrug was greater at pH 5.4 (60%) than that at pH 7.2 (12%) after 48 h. The prodrug was also able to be endocytosed by HepG2 cells and exerted effective anti-tumor effects. This responsive prodrug system also could improve the drug stability before reaching the targeting site. Wei et al. [67] also constructed a responsive conjugate by coupling DOX to a branched polymer via the hydrazone bond, which was also able to release DOX in answer to the change in pH value, thus enhancing anti-tumor efficacy in tumors with low toxicity. The group of Thurecht utilized the hydrazone bonds to couple DOX to heat shock protein 70 (Hsp70) aptamer-targeted modified HBPs, which promoted DOX accumulation in tumor cells and amplified anti-tumor effect [68].

### 2.2 pH-responsive HBPs with ionizable groups

HBPs containing abundant ionizable groups are also well suited as drug carriers for tumor treatment because of the dissociation or protonation of ionizable groups at the acidic conditions, which will alter their physical characteristics like solubility, charge, size, or solubility, and thus trigger drug release.

**Fig. 4:**
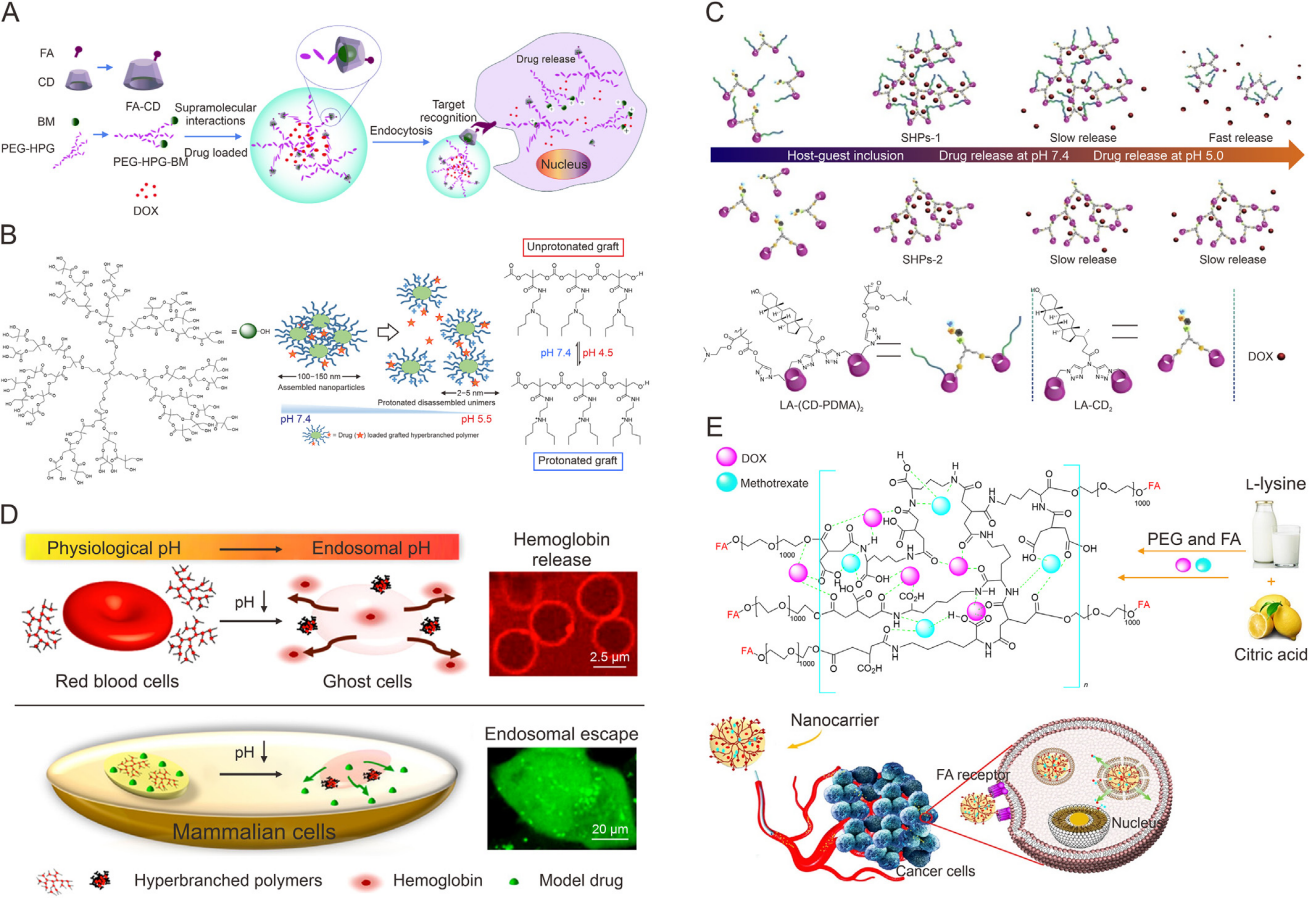
pH-responsive hyperbranched polymers (HBPs) with ionizable groups. (A) Schematic illustration of preparation of hyperbranched targeted supramolecular nanoparticles (TSNs) [70]. (B) Schematic illustration of multifunctional nanoparticles with size-transformable and pH-induced drug release from conjugated HBPs [71]. (C) Schematic illustration of the supramolecular HBPs (SHPs) self-assemble from β-cyclodextrin (CD) for drug delivery [72]. (D) Schematic illustration of the anionic poly(l-lysine iso-phthalamide) HBPs (HPLP) with pH-responsive [79]. (E) Schematic illustration of hyperbranched nano-carrier (poly(l-lysine citramide) (HBPLC)-poly(ethylene glycol) (PEG)-folic acid (FA)) for the delivery of doxorubicin (DOX) and methotrexate (MTX) [80]. BM: benzimidazole; HPG: hyperbranched polyglycerol; SHPs-1: supramolecular hyperbranched polymers 1; LA: lithocholic acid; PDMA: poly(2-(dimethylamino)ethyl methacrylate). Reprinted from Refs. [[70], [71], [72],^79^,^80^] with permission.

Amino groups are a common class of ionizable groups, which will gradually protonate with decreasing pH, resulting in changes in polymer properties. Roy and De [69] prepared a type of amino acid-based pH-responsive HBPs. The results suggested that the molecular weight of the HBPs increased linearly and the degree of branching (DB) reduced linearly with the reaction time. Because of the existence of amino groups, light transmission of the polymers gradually increased with decreasing pH, and the morphology gradually changed from micelle aggregates to stable micelles, showing good pH-responsiveness. At lower pH conditions, this property of amino groups, which changes the morphology and size of HBPs due to protonation and enhancement of hydrophilic properties, is expected to be applied in controlled drug release. Chen et al. constructed a type of hyperbranched supramolecular nanoparticles (TSNs) through the host-guest interaction of FA modified β-CD (FA-CD) and benzimidazole (BM) anchored PG (PEG-HPG-BM) (Fig. 4A) [70]. The BM moiety behaved hydrophobic under neutral conditions, whereas under acidic conditions, the polymer became more hydrophilic because of protonation of aromatic amines, which leaded to faster dissociation rate of the assemblies and increased drug release rate. The results of drug release showed that TSNs released nearly 90% DOX under acidic conditions, which was markedly larger than that under physiological conditions. Cellular experiments also showed that it could effectively promote the treatment effect of tumor, showing a promising prospect.

Tertiary amine groups are also pH sensitive. Under neutral conditions, unprotonated tertiary amine polymers can form stable aggregates, and when the pH decreases, large aggregates are likely to break down into small nanoparticles by electrostatic repulsion of amino groups, which is expected to cause the release of the drug molecules inside. For instance, Ray et al. constructed a type of size-convertible HBP nanoparticles by introducing pH-responsive tertiary amines on polyester-*b*-linear polycarbonate copolymers (Fig. 4B) [71]. Experimental results showed that the polymer self-assembled at pH 7.4 to form stable aggregates with the sizes of 150–190 nm, whereas when exposed to acidic pH, the nano-aggregates decomposed due to the protonation of the amines and decreased in diameter by a factor of about 60. Using the ability of the aggregate structure to vary in response to pH and combining this with the variability of pH inside and outside tumor cells, it is clear that this system had significant control over the release of gemcitabine (GEM), an anticancer drug embedded in or conjugated to the aggregates, with the release being much greater at pH 4.5 than that at pH 7.4. This could contribute to the selective uptake of GEM by cancer cells and inhibit the viability of pancreatic cancer cells. Additionally, this acid-mediated size change of hyperbranched nanostructures demonstrated the ability of dissociable nano-carriers to traverse dense cellular environments, showing good potential in disease treatment such as connective tissue proliferation or poorly vascularized cancer subtypes. Bai et al. adopted two β-CD-terminated poly(2-(dimethylamino)ethyl methacrylate) (CD-PDMA) and one lithocholic acid (LA) fragment to obtain an AB_2_-type LA-(CD-PDMA)_2_ macromonomer, and further prepared a HBP (PDMA) through host-guest recognition (Fig. 4C) [72]. Due to the presence of tertiary amine groups, this polymer was pH-responsive, and DOX was released more rapidly at acidic environment than that at neutral environment. This polymer could also efficiently deliver DOX to MCF-7 cells, producing a positive anti-tumor effect. The carboxylic acid group is also a common ionizable group that undergoes deprotonation under higher pH conditions, becoming charged and more hydrophilic. For example, polyacrylic acid polymer (PAA) has the properties of a polyelectrolyte material, and the carboxyl groups cause the structure of the polymer to change in the range of pH 5–6 through partial deprotonation. At lower pH, this polymer develops a tight superhelical structure; as pH increases, the carboxylic acid groups are turned into carboxylate ions and this polymer undergoes solvation to form more open helical structure [73]. Therefore, when PAA is introduced onto the HBPs, the HBPs can be given with pH responsiveness. Blackburn et al. [74] synthesized a FA and fluorescently labeled RB using 2-propylacrylic acid, disulphide diacrylate, and ethyl methacrylate as raw materials via reversible addition fragmentation chain-transfer (RAFT) copolymerization reaction. The particle size of this polymer changed from 100 nm to 397 nm when the pH altered from 7.4 to 5.4, indicating its good pH-responsiveness. After labeling with rhodamine B (RhB) dye, the polymer was found to be internalized by cells, demonstrating its potential in drug delivery. Further, Balafouti and Pispas [75] prepared a novel biocompatible hyperbranched copolymer of lauryl methacrylate, methacrylic acid, and bifunctional ethylene glycol dimethacrylate via one-step RAFT copolymerization. With the decrease of pH, due to the deprotonation of carboxyl groups, the particle size of this polymer also changed from several hundreds of nanometers to tens of nanometers, showing good pH-responsiveness. It could be loaded with hydrophobic drug curcumin (CUR) by hydrophobic interaction and be applied to drug release and bio-imaging. While Tabatabaei Rezaei et al. [76] constructed a block hyperbranched copolymer (HCAE-FA) for PTX delivery. Because of the presence of PAA in HCAE-FA, the contraction of PAA under low pH would give the nano-carrier with a nano-pump acceleration effect, which facilitated PTX release and provided an effective tumor-suppression effect.

The use of HBPs containing carboxyl groups to construct polyelectrolyte complexes has also attracted much attention. Dai et al. [77] formed polyion complex (PIC) micelles by blending sodium carboxyl surface modified hyperbranched polyester anions (Hx-COONa) with PEG-block-PCL-graft-PEI) polycations. The pH value was shown to significantly affect the DOX release because of the change of electrostatic attraction. The cumulative release after 24 h at pH 5.0 was about 80%, while that was lower than 40% at pH 7.4. Recently, Ahmed et al. [78] also obtained a stabilized PIC by electrostatic interaction of hyperbranched polyamido amines (PAMAMs) with SA modified zwitterionic chitosan (ZWC) at physiological pH. When pH was lower than 7, because of protonation or deprotonation of amino or carboxyl groups, the ZWCs would undergo charge reversal and exhibit electropositive surfaces, which lead to the disaggregation of the PIC because of the electrostatic repulsive force, which in turn trigger the release of 5-FU. Thus, this system has excellent prospects in tumor therapy.

Amino acid compounds are also commonly used to prepare pH-responsive hyperbranched polymeric carriers because of their good biocompatibility and naturally ionizable groups. For example, Wang and Chen prepared anionic, pH-responsive, poly(l-lysine iso-phthalamide) HBPs (HPLP) with different DB, which mimicked the function of cell-penetrating peptides for effective drug delivery through a simple one-pot synthesis method (Fig. 4D) [79]. Under physiological conditions, the polymer was electronegative, had good hydrophilicity and no membrane-penetrating ability, and exhibited non-hemolytic activity. Under acidic conditions, the polymer would gradually transform to hydrophobic due to protonation of the carboxyl groups, resulting in a significant boost in membrane-lytic activity. The results showed that the HBPs could efficiently delivery the calcein into the lysosome, thus cause disruption of the lysosomal membrane, thereby effectively releasing calcein into the cytoplasm. Therefore, these HBPs show good potential for intracellular delivery of biomolecules. In addition, Aslani and Namazi also synthesized a hyperbranched poly(l-lysine citramide) (HBPLC) by polycondensation method using l-lysine and citric acid as raw materials, and further functionalized it by PEG and FA to construct a nano-carrier (HBPLC-PEG-FA) for the delivery of DOX and methotrexate (MTX) (Fig. 4E) [80]. It was found that loading rates of DOX and MTX could reach 96.3% and 96.9%, respectively, because of strong interactions inside this carrier. The drug release also showed significant pH-responsiveness. For example, under acidic conditions, cumulative DOX release at 72 h was 92.7%, much higher than that in neutral conditions (64.5%), and cumulative MTX release at 144 h was 97.02%, also much higher than that at the usual physiological pH (60.6%). Cellular experiments showed that the carrier had good biocompatibility but strong cytotoxicity after drug loading. The half maximal inhibitory concentration (IC_50_) against MCF-7 cells was only 55 μg/mL. The group further introduced glucose-derived quantum dots (GluQD) [81] and Fe_3_O_4_ nanoparticles [82] into the HBPs, thus further endowing them with photoluminescence and magnetic responsiveness and expanding their applications in tumor therapy.

Recently, Xia et al. [83] synthesized a type of hyperbranched polyether with full pH-range responsiveness. They firstly synthesized hydrophobic poly(3-methyl-3- (hydroxymethyl)oxetane) HBPs (HPMHO). Further modification of the terminal hydroxyl groups of HPMHO by ethylenediamine (EDA) or succinic anhydride (SA) gave a suite of amphiphilic amine-modified HPMHO and carboxyl-modified HPMHO, respectively. They could all self-assemble into micelles, show pH-responsive phase transition properties, and the amount of surface modification could be controlled by adjusting the amount of EDA or SA dosed, further regulating the pH-responsive range. These hyperbranched polyether also have advantages in biocompatibility and have promising applications in the area of drug carriers.

### 2.3 pH-responsive HBPs modified hybrid nanomaterials

Natural macromolecular materials such as proteins and polysaccharides have the advantages of good biodegradability, biocompatibility, and hypotoxicity, and have gained much interest in drug delivery, but they often have the disadvantages of complex and inefficient surface functionalization methods and low drug loading. Therefore, the effective integration of HBPs and natural macromolecular carrier materials can maximize the advantages of both, and thus the construction of multifunctional hybrid nano-drug delivery systems is of great significance.

**Fig. 5:**
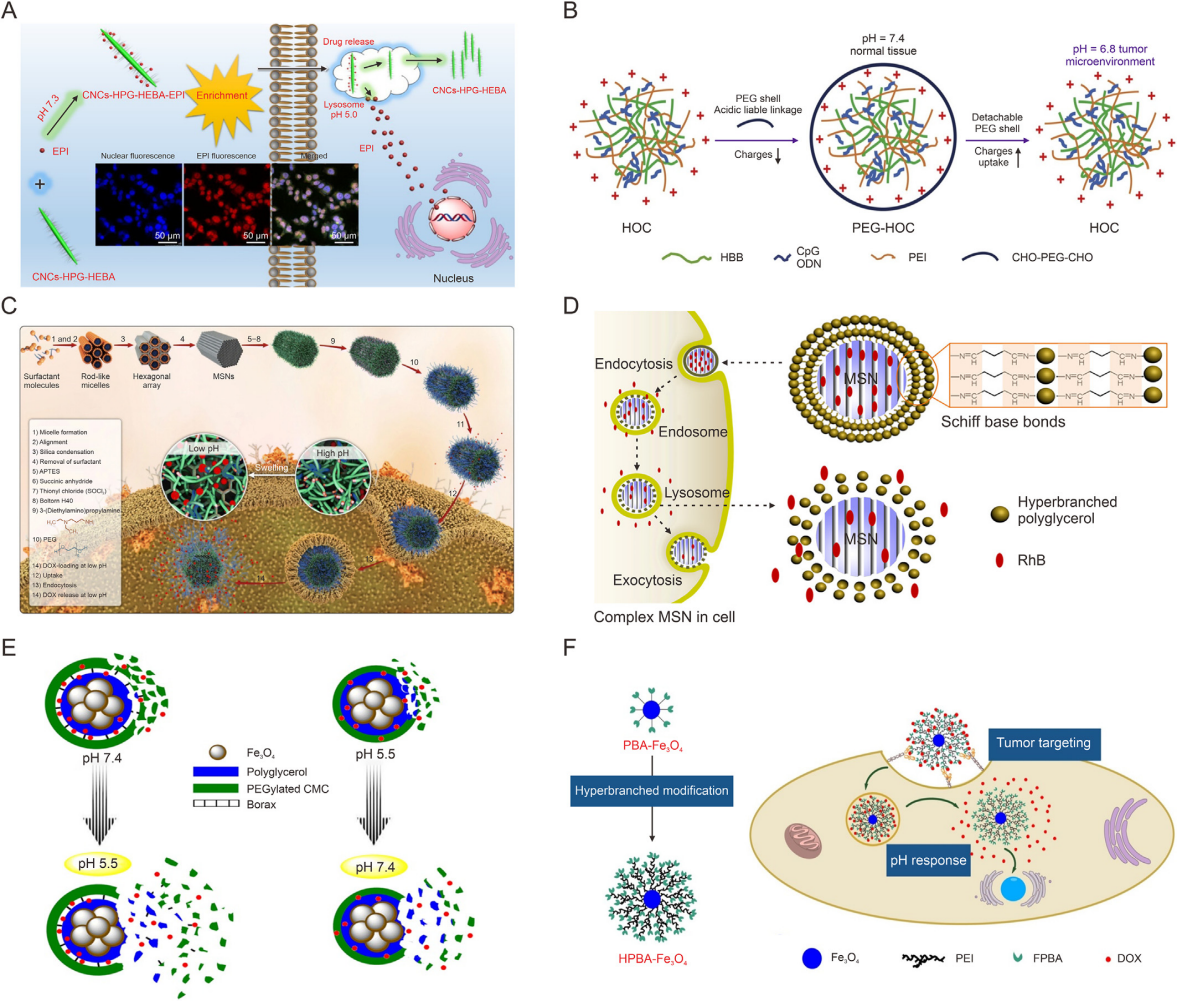
pH-responsive hyperbranched polymers (HBPs) (RBs) modified hybrid nanomaterials. (A) Schematic illustration of the HBP-functionalized cellulose nanocrystals (CNCs) with surface hydrazone bond-linked epirubicin (EPI) [84]. (B) Schematic illustration of the hyperbranched dextran (HBB) based polyelectrolyte complex (HBB/polyethyleneimine (PEI)/cytosine-phosphate-guanine (CpG) (HOC)) for cytosine-phosphate-guanine oligodeoxynucleotide (CpG ODN) delivery [85]. (C) Schematic illustration of the preparation, cellular uptake and doxorubicin (DOX) release of rod mesoporous silica nanoparticles (MSNs) based pH-responsive hybrid carrier (PEG-Boltorn H40 (H40)-MSNs) [90]. (D) Schematic illustration of the uptake and release of guest molecules from the acid-responsive hyperbranched PG (HPG)-MSNs carriers [91]. (E) Schematic diagram of the borate ester bonds crosslinked HPG and carboxymethyl cellulose based magnetic carriers for the delivery of DOX [94]. (F) Schematic illustration of the 4-formylphenylboronic acid (FPBA) group modified hyperbranched magnetic Fe_3_O_4_ nanoparticles (HPBA-Fe_3_O_4_) for tumor-targeting treatment with pH responsiveness [97]. HEBA: hydrazidation ethyl bromoacetate; PEG: poly(ethylene glycol); CHO: aldehyde; APTES: 3-aminopropyltriethoxysilane; RhB: rhodamine B; PBA: phenylboronic acid. Reprinted from Refs. [84,85,90,91,94,97] with permission.

Cellulose nanocrystals (CNCs) have great potential in biomedical areas due to their favorable physicochemical properties, included high strength, excellent biodegradability, renewability, etc.. However, the complex surface functionalization and low yield of CNCs lead to difficulty for the drug loading to reach an effective concentration that inhibits tumor cells, thus limiting their further development in drug delivery. Surface functionalization of CNCs using HBPs is expected to present numerous of active end groups on them, thereby avoiding their shortcomings. Thus, Wan et al. prepared a HBP-functionalized CNCs (CNCs-HPG) directly by anionic polymerization of glycidol utilizing acidified CNCs as initiator (Fig. 5A) [84]. Terminal hydroxyl was then converted into hydrazide bond that would form acid-sensitive hydrazone bond with carbonyl group on the broad-spectrum anti-tumor drug EPI, thus a RB prodrug was constructed. The introduction of HBPs not only further improved the dispersion of CNCs, but also improved the functionalization of the carrier and increased the loading capacity of EPI by taking advantage of the increased active sites of the polymers, so that the DLE of EPI reached 42.45%. The EPI release at acidic environment was also much higher than that at normal condition. And the system was efficiently internalized by Huh7 cells while EPI maintained its biological activity to inhibit tumor cell growth. The successful synthesis of this pH-responsive heterogeneous class of carriers has opened up a new direction in the design of multifunctional polymers for CNCs and broadened the scope of applying functional HBPs in the area of drug delivery.

Dextran, another bio-renewable natural carbohydrate material, has excellent biodegradability and biocompatibility, and can provide many reaction sites for the formation of nano-carrier particles and the protection/encapsulation of drugs. Therefore, dextran also has a great potential in drug delivery. Su et al. used positively charged PEI, hyperbranched dextran (HBB), negatively charged cytosine-phosphate-guanine (CpG), and CPG oligodeoxynucleotide (ODN) to form a polyelectrolyte complex (HBB/PEI/CpG (HOC)) (Fig. 5B) [85]. On this basis, aldehyde-modified PEG was introduced to bind to the amino groups of PEI through Schiff base bond, which not only reduced toxicity, but also allowed the polymer carrier to shed the PEG shell via pH-responsiveness in acidic tumor microenvironment, promoting the intracellularization of HOC, thus further enhancing the therapeutic efficacy. On the other hand, Qin et al. [86] combined peptides and polysaccharides with pH-responsive HBPs to enhance biocompatibility as well as physiological activity. The authors first synthesized poly(diisopropylaminoethyl methacrylate) (hPDPA) HBPs, whose backbone contained the amantadine groups. β-CD modified polyarginine peptide (β-CD-PArg) was then attached using host-guest interactions, which in turn led to hyaluronic acid (HA) adsorption on its surface through electrostatic interaction. Finally, the obtained hybrid polymer could self-assemble into vesicles under neutral conditions, which showed low cytotoxicity to HeLa cells. It could effectively encapsulate β-lapachone (β-lapa) and release β-lapa quickly under acidic conditions (pH 5.0). β-lapa could increase the intracellular ROS level and produce nitric oxide (NO) by reacting with PArg, resulting in a synergistic anti-tumor effect.

Inorganic/organic hybrid nano-carriers have also attracted great attention, which combine the biocompatibility and degradability of organic compounds with the stability of inorganic nano-carriers. Among these, mesoporous silica nanoparticles (MSNs) have high stability, high drug loading potential, and good biocompatibility, and thus play a key part in delivering drugs [[87], [88], [89]]. The existence of vast surface hydroxyl groups of silica also makes it possible to anchor a wide range of materials, thus further improving its properties. Bafkary et al. modified the surface of rod MSN with H40 hyperbranched polyester and further connected the amine-modified PEG at the hydroxyl terminal of H40, thus a pH-responsive hybrid carrier (PEG-H40-MSN) was obtained for DOX delivery (Fig. 5C) [90]. Because of the porous structure of MSN, the DOX loading was greatly improved. Moreover, under normal physiological conditions, the polymer shell contracted and were neutral, thus DOX was better loaded in the microporous pores, obtaining the loading rat was 36.5% and the encapsulation rate was 57.4%, whereas under low pH, the polymers were positively charged and repelled each other due to the protonation of the tertiary amines on H40, which resulted in the swelling of the shell and led to the release of DOX. An *in vitro* release experiment shown that the cumulative DOX release within 48 h was 9.7% and 20% at normal pH and acidic pH (6.8), respectively, while at pH 5.5, it was remarkably increased to 49%, which fully demonstrated the pH-dependence of DOX release by PEG-H40-MSN. More importantly, the hemolytic activity of PEG-H40-MSN was less than 1.6% in a wide concentration scope, indicating this carrier had good biocompatibility.

Yang et al. constructed acid-responsive carriers (HPG-MSNs) by coupling HPG to the surface of MSNs via imine linkages (Fig. 5D) [91]. Studies using RhB as an object molecular model revealed that the release of RhB was very slow at normal pH, whereas it was fleetly increased at pH 5.5 due to the breakage of imine linkages. The carrier also had low cytotoxicity and could be endocytosed by tumor cells, making it a potential carrier in tumor therapy. Similarly, Li et al. [92] successfully synthesized a type of multifunctional MSN (MSN-COOH-tetrandrine (Tet)-HBP-FA) to serve as a carrier for the anti-tumor drug Tet. The carboxyl group functionalization on the support surface increased its adsorption capacity of Tet up to 26.86%. The terminally aminated HBP was attached to the MSN through amide condensation with the carboxyl and encapsulated the drug molecules within the pores, which improved the carrier stability and allowed to realize “zero pre-release” within the first 20 h in the physiological environment. The carrier showed pH-responsiveness to drug release, which was expedited under acidic media by the dissociation of HBP, leading to the exposure of small pores of MSN. It was shown that the system released 30% within 72 h in pH 4.5, while it was only 10% in pH 7.4. In addition, due to the surface modification of FA, the system was well biocompatible for lysosomal uptake of A549 and HeLa cells, and exhibited active FA receptor-mediated uptake, which increased the cell internalization of the drugs. These suggest that HBP-functionalized MSNs have strong stability and high drug-carrying capacity and are potential carriers of anticancer drugs.

Organic-inorganic core-shell nanostructures composed of magnetic Fe_3_O_4_ are widely explored hybrid nanomaterials and have attracted great interest among researchers [93]. For instance, Zohreh et al. obtained a borate ester bonded nano-carrier with magnetic Fe_3_O_4_ nanoparticle as core, hyperbranched PG as the first shell, and DOX and PEGylated carboxymethylcellulose as the second shell (Fig. 5E) [94]. The results show that DLC and DLE of DOX could reach 240.4 mg/g and 48.08%, respectively. The release of DOX also showed pH-dependence, with faster release at low pH. For example, after 80 h, the cumulative release at pH 5 was 74%, while it was only 34% at the physiological pH. The reason for this was due to the fact that the borate eater bonds formed by borax crosslinking agent was prone to breaking under acidic conditions, which reduced the degree of crosslinking of the shell layer, thus leading to accelerated drug release. The HBP-modified nanosystems also produced significant inhibitory effects on HeLa cells. In addition, it had the transverse relativity *r*_2_ effect from magnetic Fe_3_O_4_ that is expected to realize visual treatment of tumor. Moreover, Tabatabaei Rezaei et al. [95] designed a pH-sensitive hyperbranched quercetin delivery system (Fe_3_O_4_@PAMAM-b-PEG-FA). The release of quercetin may be stimulated by acidic pH changes, thus reducing the non-specific diffusion in blood circulation. It could also selectively accumulate into tumor cells through FA receptor-mediated endocytosis, enhancing intracellular drug level and anti-tumor influence. In addition, even with low iron content, this magnetic nano-carrier showed high magnetic resonance imaging (MRI) sensitivity [96].

In other work, Song et al. coupled hyperbranched PEI onto Fe_3_O_4_ nanoparticles and further exploited the reaction of terminal amino with 4-formylphenylboronic acid (FPBA) to obtain a type of PBA group modified magnetic nanoparticles (HPBA-Fe_3_O_4_) (Fig. 5F) [97]. Due to the presence of PBA ester bonds, HPBA-Fe_3_O_4_ could greatly improve the DOX loading as well as stability, which could release on account of the breakage of the boronic acid ester bonds in micro-acidic condition. Results showed that release at pH value of 5.0 was almost twice that at pH 7.0 after 72 h *in vitro*. Meanwhile, since PBA could bind to sialic acid on the cell surface, HPBA-Fe_3_O_4_ was tumor-targeted, which could enhance the concentration of DOX in U-87 MG cells, and produce a stronger anti-tumor activity than free DOX.

Carbon nanomaterials, such as graphene and carbon nanotubes, have also shown satisfactory advantages in drug carriers thanks to their huge specific surface and oxygen-rich multifunctional groups [98]. Consequently, Lyra et al. [99] employed guanidinylated derivatives of hyperbranched PEI on multiwall carbon nanotubes (MWCNTs) modification to obtain a novel water-soluble hybrid drug delivery carrier (oxCNTs@GPEI). It could enhance the loading of DOX which could be as high as 99.5%, and it showed pH-responsiveness to the DOX release. In comparison with pure DOX, the encapsulation of the carrier could enhance the internalization of DOX and present stronger cytotoxicity. While Pourjavadi et al. [100] constructed a graphene oxide (GO) based carrier, in which DOX was linked with pH-responsive acylhydrazine bond and CUR was loaded by π−π stacking. Cumulative release rates of CUR at the pH value of 7.4 and 5.0 were approximately 15% and 26% within 73 h, respectively, which was mainly due to the weakening of the interaction between CUR and GO under acidic conditions [101], whereas the cumulative DOX release, which profited by the breaking of acylhydrazone bond, were 35% and 87%, respectively, indicating that the release of both showed a pH-responsiveness. Cellular experiments also showed that DOX and CUR were able to produce synergistic anti-tumor effects and improve therapeutic efficacy. In addition, GO had a photo-thermal conversion effect, which could also produce a certain tumor therapeutic ability, thus improving the anti-tumor efficacy [102].

## 3 Redox-responsive HBPs

Compared to normal cells, tumor cells have high levels of redox and excessive oxidative stress. The dysregulation of redox balance due to disturbed levels of reduced glutathione (GSH) [103] and ROS [104] in tumor cells enhances the resistance of tumor cells to oxidative stress, causing resistance to chemotherapeutic drugs. Therefore, the redox environment within tumor cells is a potential design target for responsive polymers [[105], [106], [107]]. To take full advantage of this feature, responsive nano-delivery systems based on redox levels have been extensively investigated, mainly focusing on aspects such as the design of ROS- or GSH-responsive compounds or backbone structures. Here, we analyzed the design principles of both classes of responsive HBPs and the principles of controlling drug delivery to explore their potential in the treatment of cancer.

### 3.1 ROS-responsive HBPs

ROS are the by-product of biological aerobic metabolism and include hydroxyl radicals (∙OH), superoxide anions (O_2_∙^−^), hydrogen peroxide (H_2_O_2_), etc.. ROS play a crucial part in maintaining the redox balance of human physiological functions, cellular conductance, and protein synthesis under normal physiological conditions. However, ultra-high levels of ROS can directly break down protein, lipid, and nucleic acid molecules, triggering oxidative stress. Meanwhile, high ROS levels have been found in clinical studies that may both cancerate normal cells and further promote tumor cell infiltration and metastasis [104]. In particular, the concentration of H_2_O in tumor tissues (0.1 × 10^−3^−1 × 10^−3^ mM) is noticeably over that of normal tissues (35 × 10^−6^ mM), and this is closely related to tumorigenesis and progression. It is this difference in ROS levels that promotes the development of ROS-responsive polymers [[108], [109], [110]]. Therefore, the introducing ROS-responsive structures or groups, such as phenylborate ester (PBE), ferrocene (Fc), thioether, and their derivatives, etc., will enable the HBPs to release drugs in reaction to the elevated ROS levels of cancer cells.

**Fig. 6:**
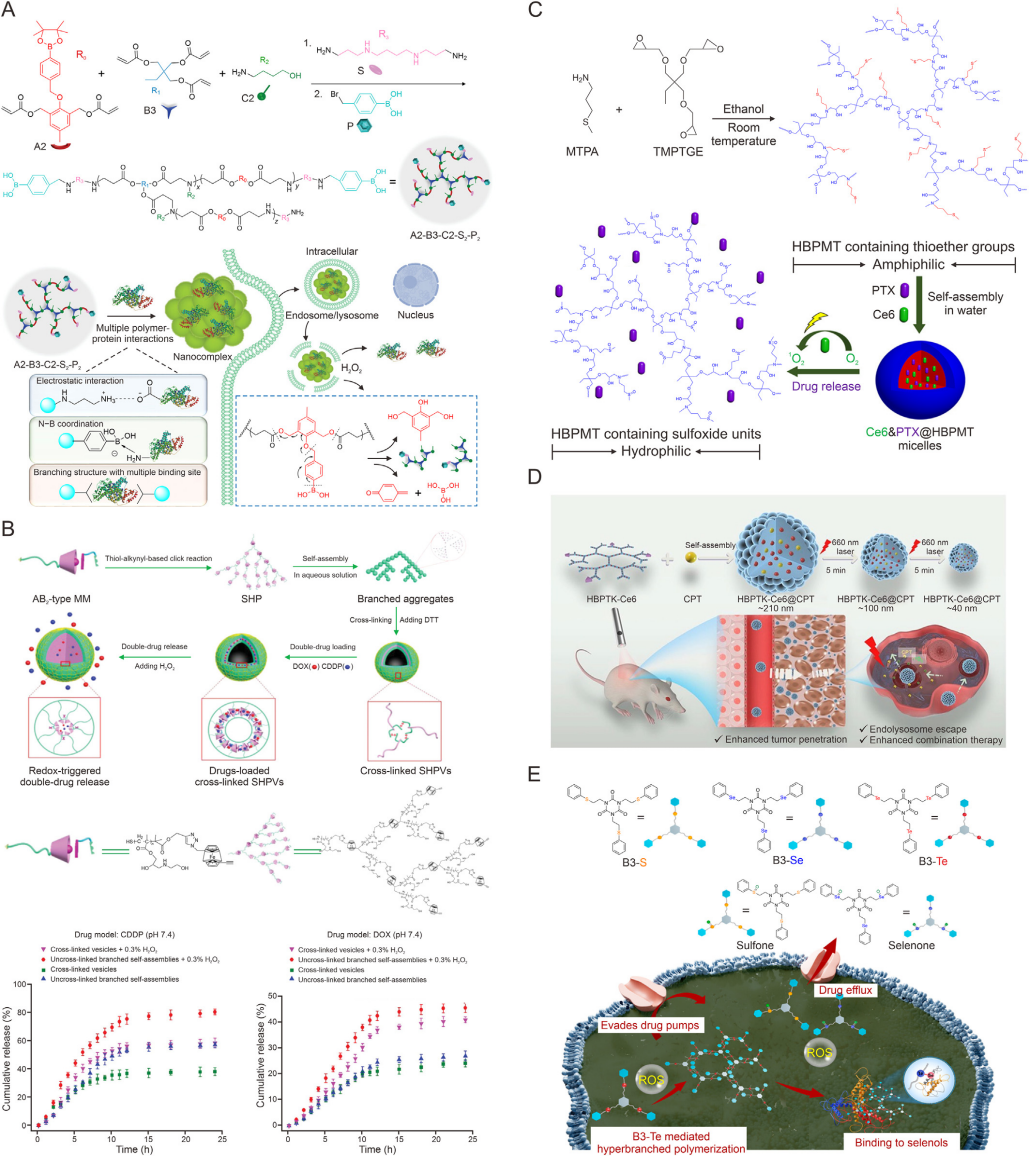
Reactive oxygen species (ROS)-responsive hyperbranched polymers. (A) Schematic illustration of the poly(β-amino ester) HBPs (HPAEs) with phenylboronic acid (PBA) in the backbone for delivery of natural proteins [112]. (B) Schematic illustration of the design of redox-responsive HBP vesicles (SHPVs)) and the controlled release curves of doxorubicin (DOX) and *cis*-platinum (CDDP) dual drugs [116]. (C) Schematic illustration of the amphiphilic HBP containing thioether (HBPMT) for the release of paclitaxel (PTX) and chlorin e6 (Ce6) [120]. (D) Schematic illustration of hyperbranched thioketal and Ce6-based polyphosphoester (HBPTK-Ce6) for loading and delivering camptothecin (CPT) [123]. (E) Schematic illustration of the *in-situ* synthesis of HBPs in living cells by ROS-induced oxidative polymerization of organotelluric compounds [125]. MM: macromonomer; SHP: supramolecular hyperbranched polymer; DTT: dithiothreitol; MTPA: 3-(methylthio)propylamine; TMPTGE: trimethylolpropane triglycidyl ether. Reprinted from Refs. [112,116,120,123] with permission.

PBE is one of the common ROS-reactive functional groups: in addition to its pH responsiveness, it is also highly selective and sensitive to H_2_O_2_. Thus, phenylboronic esters have good application prospects in the bio-nano field. On one hand, PBE can act as a hydrophobic fragment in the constructed drug carriers, responding to ROS and triggering carrier degradation and drug release [111]; on the other hand, when PBE is modified on the surface of a polymer carrier, it can selectively interact with sialic acid that high expressed on the surface of tumor, thus making it targeted. For example, Liu et al. designed a kind of poly(β-amino ester) HBPs (HPAE) with PBA within the backbone via a facile polycondensation reaction for effective delivery of natural proteins (Fig. 6A) [112]. The PBA embedded in the polymer backbone not only enhanced binding to proteins through N–B coordination, resolving the contradiction between over-encapsulation of protein drugs and insufficient intracellular release, but also exhibited H_2_O_2_ sensitivity. When this polymer is in contact with high concentrations of H_2_O_2_ inside tumor cells, the PBE inside will oxidize and decompose, and the rearrangement via quinone carboxamide trigger the backbone breakage and degradation, thus not only accelerating protein release, but also reducing the toxicity of polycationic materials. Moreover, through adjusting DB, the charge density of cations, and the spatial distribution of PBE, the carrier could also effectively deliver a variety of natural drugs, which may hold a critical position in the fields of chemotherapy, gene editing, and other fields.

Zhang et al. [113] constructed a ROS-responsive HBP with metallisable 8-hyroxyquinoline (HQ) units based on iminoboronate (IB) backbones (HBP(oligoethylene glycol (OEG-IB)-HQ). Compared with normal cells, the concentration of Cu(II) ions inside cancer cells is larger, so HBP(OEG-IB)-HQ was able to bind intracellular Cu(II) in tumor cells to form micelle aggregates, which generated more ROS through a Fenton-like reaction with intracellular H_2_O_2_, thereby triggering the cleavage of the IB and the dissociation of the carriers, which in turn induced the rapid release of camptothecin (CPT). It was shown that the release was significantly faster than that of the control, which was expected to produce more significant anti-tumor effects. This approach increased intracellular ROS levels, thereby enhancing the sensitivity of ROS-responsive polymers and extending the therapeutic effect.

Fc and its derivatives are also ROS-responsive. Under normal physiological conditions, Fc is a hydrophobic and neutral; while under a specific microenvironment with high ROS level, it will be converted to hydrophilic ferrocenium ions (Fc^+^) through oxidation, which may break the structure of the polymeric carriers and achieve drug release [114]. In addition, the 3D structure of Fc can form redox-responsive host-guest structures with various macrocyclic host compounds, such as cyclodextrins, cupric aromatic hydrocarbons, and cucurbiturils, and so it has been widely used in the preparation of redox-responsive supramolecular complexes. For instance, Chen et al. [115] constructed a hyperbranched assembled drug delivery system with NO- and redox-stimulated responsiveness by orthogonal self-assembly strategy based on β-CD functionalized organoplatinum(II) metallacycle and three-arm PEGylated Fc. Its internal cavity could be used to load DOX, and when exposed to NO or H_2_O_2_, its spherical structure would be disrupted because of the detachment of Fc to release more platinum and DOX, thus exerting an effective synergistic anti-tumor effect. Du et al. prepared a sort of redox-sensitive hyperbranched supramolecular polymer vesicles (SHPVs) through host-guest recognition of Fc and β-CD for delivering *cis*-platinum (CDDP) and DOX (Fig. 6B) [116]. With the addition of H_2_O_2_, Fc could be oxidized to Fc^+^, leading to the destruction of host-guest interaction, which in turn induced the rapid release of CDDP and DOX.

Thioethers are also redox-responsive and are readily oxidized to more hydrophilic sulphoxide or sulphone under the action of ROS, completing the hydrophobic to hydrophilic phase transition, and thus can also be used to construct carriers for delivering oncology drugs [117]. For instance, Wu and Liu [118] prepared a sort of aliphatic poly(β-thioether ester)s hyperbranched nanoparticles. It was found by ultraviolet (UV) spectroscopy that the transmittance of this prepared nano-solution could change quite sluggish when the H_2_O_2_ concentration was 1% (*m*/*V*), whereas at 3% (*m*/*V*) of H_2_O_2_, a significant transition from cloudy to transparent would be found in 4 h. Moreover, the transmittance showed a concentration-dependent dependence on H_2_O_2_, because of the conversion of thioether units into sulfoxides or sulfones through oxidation reactions. All of the above indicated that poly(β-thioether ester)s have ROS-responsive properties and also suggest their potential in drug release. Liu et al. [119] further prepared a 7-ethyl-10-hydroxy-CPT (SN38)-linked HPG (HPG-2S-SN38) through thioether linkage. It was able to assemble to form micelle and encapsulate cinnamaldehyde (CA) which can induce cell death by producing ROS. The drug-loaded micelles could enter into the tumor cells and rapidly release SN38 and CA under the action of ROS via thioether fracture. Meanwhile, the preemptively released CA could generate abundant ROS, which further accelerates micelle dissociation and drug release, resulting in an anti-tumor synergistic effect. Moreover, Wang et al. constructed a thioether based hyperbranched amphiphilic polymer (HBPMT), which self-assembled into a stable micelle for encapsulation of PTX and Ce6 (Fig. 6C) [120]. In the presence of light, Ce6 in this micelle could generate ROS, which leads to the breakage of thioether in the micelle nucleus, inducing micelle decomposition and PTX release. This type for *in situ* generation of large amounts of ROS by external stimuli can enhance the sensitivity of HBPs, which not only achieves controlled release of drugs, but also holds the promise of synergistic chemo-photodynamic anti-tumor effects. Similar to thioether, thioketal is also ROS-responsive and is notable in the field of constructing responsive carriers due to its biodegradability and high safety [121]. For example, Sun et al. [122] constructed a singlet oxygen (SO)-responsive hyperbranched polyphosphates with thioketal linkers (RHPPE) for the simultaneously delivery of Ce6 and DOX through [A_2_+B_3_]-type polycondensation. When exposed to 660 nm light, Ce6 could produce vast SO, which triggers the death of tumor cells and also causes thioketal rupture, thus accelerating the release of DOX, leading to a synergistic anti-tumor effect. Similarly, Jin et al. also prepared a thioketal and Ce6 based amphiphilic hyperbranched polyphosphoester (HBPTK-Ce6) for loading and delivering CPT (Fig. 6D) [123]. In the presence of 660 nm light, Ce6 mediated generation of ROS led to thioketal breakage and the size of the nano-carrier gradually decreased, thus accelerating the release of CPT, which could also synergize with Ce6.

In recent years, organotelluric polymers with reversible redox properties have also gradually come to the front [124]. These systems can respond efficiently to intracellular ROS to initiate oxidative polymerization in specific environments. On one hand, we can exploit the redox characteristics of organotellurium to achieve responsive drug release in tumors. On the other hand, we can combine the broad target surface area and multilayered branching structure of HBPs in their own structure to resist the efflux effect. To make full use of these advantages, Liu et al. proposed the *in-situ* synthesis of HBPs in living cells by ROS-induced oxidative polymerization of organotelluric compounds to achieve selective anti-tumor and avoid toxic effects on normal cells as much as possible (Fig. 6E) [125]. The authors delivered B3−Te monomers with highly sensitive to ROS into cells, which would polymerize *in situ* in response to ROS to form hyperbranched nanostructures over time. The Te−O structures in this HBP may affect the cellular antioxidant system by inhibiting thiol activity in selenoproteins. It was shown that it could lead to a decrease in protein expression of GSH peroxidase 4 (GPX4). In addition, this hyperbranched structure facilitated resistance to the exocytosis effect of drug pumps in cells, prolonging the duration of the action and improving anti-tumor efficacy. This strategy of selective *in-situ* synthesis of HBPs in tumor cells provides an important reference direction for avoiding drug resistance in tumor cells.

### 3.2 GSH-responsive HBPs

GSH is an important reducing molecule within cells, and the sulfhydryl group of cysteine in its structure is the main functional group of the compound. Catalyzed by GPX, GSH is able to reduce intracellular H_2_O_2_ and convert it to water. Due to the overproduction of toxic ROS in tumor tissues, cells thus need to produce excess GSH to counteract ROS-induced oxidative stress. Therefore, the construction of GSH-responsive HBPs for anti-tumor drug delivery is encouraging. Among them, the introduction of disulfide bonds into HBPs is one of the common strategies to prepare GSH-responsive HBPs [126,127]. The disulfide bond-based drug delivery system of HBPs can remain stable in circulation, thus avoiding premature drug release and reducing systemic toxicity; while when it is internalized by the tumor cells, large concentrations of GSH will cause the breakage of disulfide bonds, triggering the degradation of the carrier and accelerating the release of the contained drugs.

A common approach to prepare HBPs containing disulfide bonds is to use the polymerization reaction of monomers containing disulfide bonds to introduce them directly into the backbone or side chains of the polymers. For instance, Bej et al. [128] designed a type of bioreducible hyperbranched polydisulphides using [A_2_+B_3_] condensation method using aliphatic thiols and pyridinyl disulphides as A and B functional groups, respectively. The mild reaction conditions of this method led to the synthesis of HBPs with intermediate molecular weight, low dispersibility, and high DB, which allowed effective post-polymerization functionalization without any other functional groups, providing ideas for the effective synthesis of hyperbranched polydisulphides. Similarly, Chen et al. [129] synthesized a kind of PEGylated hyperbranched polyphosphoesters (DOX@ss-hbPPE) with the crosslinking of disulfide bonds for effectively encapsulating of DOX. It exhibited excellent redox-responsive drug release behavior. In the existence of reduced dithiothreitol (DTT), DOX@ss-hbPPE increased in size from ∼80 to ∼220 nm and cumulative DOX release of was 50% within 72 h. While without DTT, the nano-size change was smaller and the release was only 23%. It indicated that disulfide bonds in this system could be cleaved in response to reducing substances and accelerates drug release. In addition, this nanoparticle could be effectively absorbed by breast cancer cells and rapidly achieve DOX release inside the cells, which indicated that it was a powerful drug delivery system.

**Fig. 7:**
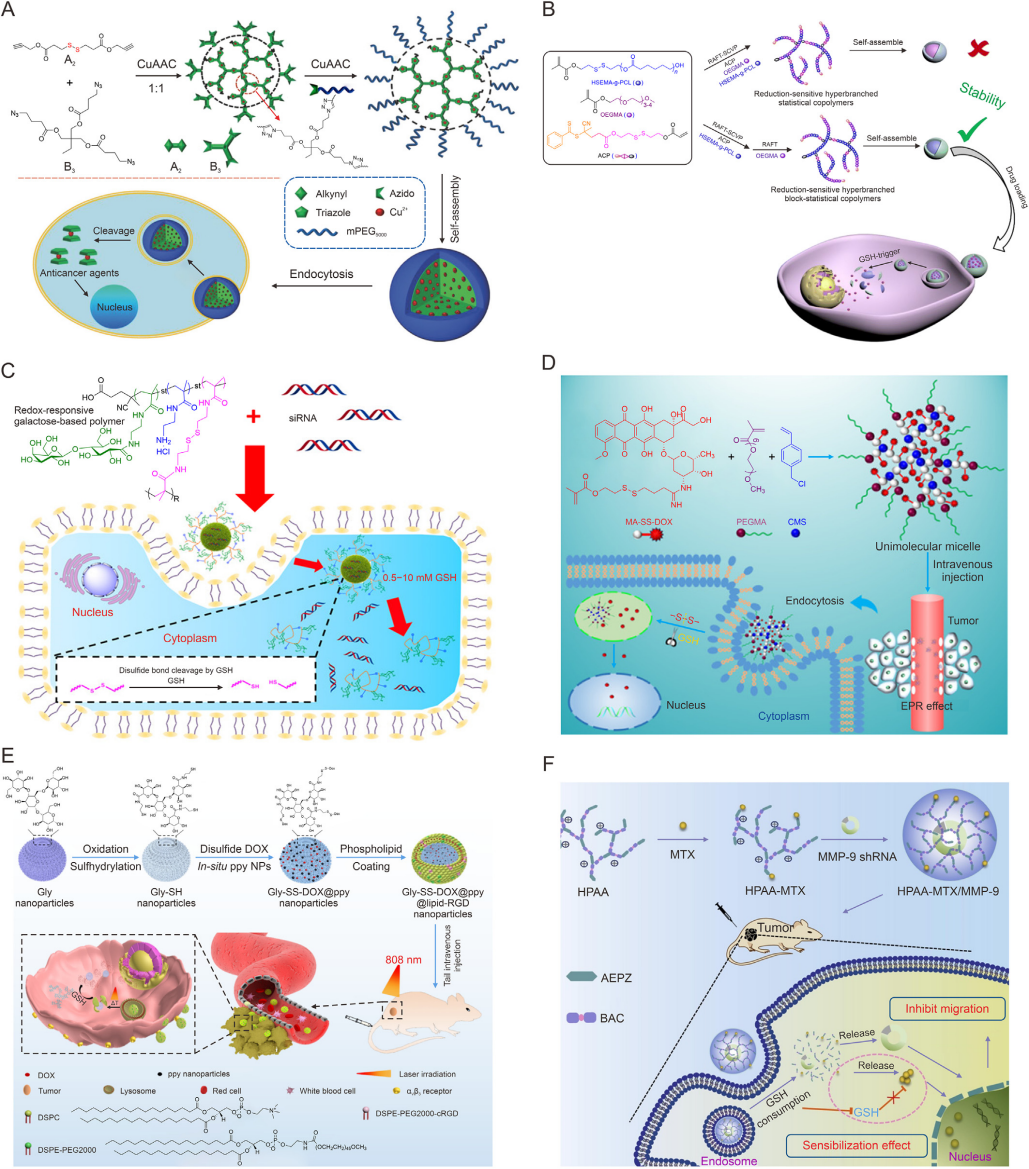
Glutathione (GSH)-responsive hyperbranched polymers (HBPs). (A) Schematic illustration of the preparation of hyperbranched Cu-based polytriazoles ((methoxy poly(ethylene glycol)) (mPEG)-hyperbranched (hb)-S-S-polytriazoles (PTAs)) [130]. (B) Schematic illustration of the biodegradable block-statistical HBPs (h-P(2-((2-hydroxyethyl)disulfanyl)ethyl methacrylate (HSEMA)-g-poly(ε-caprolactone) (PCL))-b-poly(oligo(ethylene glycol) methacrylate) (POEGMA)) for the delivery of doxorubicin (DOX) [133]. (C) Schematic illustration of the galactose-based HBPs with redox-responsive (HRRP) for gene delivery [136]. (D) Schematic illustration of redox-sensitive HBP unimolecular micelle precursor (HBPP-DOX) for the tumor-selective drug delivery [140]. (E) Schematic illustration of the hyperbranched lipid-decorated glycogen (Gly) nanoparticles (Gly-SS-DOX@polypyrrole (ppy)@lipid-arginine-glycine-aspartic acid (RGD)) for synergetic chemophotothermal tumor treatment [144]. (F) Schematic illustration of the hyperbranched poly(amido amine) (HPAA) based methotrexate (MTX)/metalloproteinase-9 (*MMP-9*) co-delivery system (HPAA-MTX/MMP-9) for tumor treatment [154]. CuAAc: Cu(I)-catalyzed azide-alkyne cycloaddition; OEGMA: oligo(ethylene glycol) methacrylate; ACP: 2-((2-(acryloyl oxy)ethyl)disulfanyl)ethyl 4-cyano-4-(phenylcarbonothioylthio) pentanoate; RAFT: reversible addition-fragmentation chain transfer polymerization; SCVP: self-condensing vinyl polymerization; siRNA: small interfering RNA; MA: ethyl methacrylate; PEGMA: poly(ethylene glycol) methacrylate; CMS: p-chloromethylstyrene; EPR: enhanced permeability and retention effect; DSPC: 1,2-distearoyl-*sn*-glycero-3-phosphocholine; PEG: polyethylene glycol; cRGD: cyclic Arg-Gly-Asp peptide; DSPE: 1,2-distearoyl-*sn*-glycero-3-phosphoethanolamine; shRNA: hairpin RNA; AEPZ: 1-(2-aminoethyl) piperazine; BAC: *N*,*N*′-*bis*(acryloyl) cystamine. Reprinted from Refs. [130,133,136,140,144,154] with permission.

Ban et al. prepared hyperbranched Cu-based polytriazoles (mPEG-hb-S-S-PTAs) through step-wise [A_2_+B_3_] polymerization reaction (Fig. 7A) [130]. The use of the disulfide-based A_2_ monomers enabled the final polymer to release large amounts of copper-triazole coordination complexes within the tumor cells, leading to a significant anti-tumor effect. In addition, the polymers exhibited an aggregation-induced emission (AIE) enhancement, showing good prospects in the field of integration of diagnosis and treatment. They also synthesized a disulfide-bonded hyperbranched PCL based star polymers for controlled release of DOX using similar reaction, which also showed a bright future [131]. Moreover, to increase DOX loading, Chen et al. [132] firstly prepared a class of disulfide-bonded reduction-cleavable HBPs (ds-HP-alkyne) through azide-alkyne [3+2] cycloaddition. The authors continued to take ds-HP-alkyne as monomer and DOX as the template, and obtained a molecularly imprinted polymer of DOX (MIP-DOX-HP), which could greatly improve DOX loading properties and release adjustability.

In the synthesis of redox-responsive HBPs, the control of the DB is very important. Therefore, Zhang et al. synthesized a reducible macromonomer containing disulfide bonds (HSEMA-g-PCL), which enabled the efficient synthesis of a type of biodegradable block-statistical HBPs (h-P(HSEMA-g-PCL)-b-poly(oligo(ethylene glycol) methacrylate) (POEGMA)) through the polymerization with oligo(ethylene glycol) methacrylate (OEGMA) (Fig. 7B) [133]. The preparation was simple and easy to perform, and the obtained HBP was able to form micelle and encapsulate DOX. In an environment with high level of GSH, this drug delivery carrier dissociated and released DOX, which was able to reach a cumulative release rate of 66% after 72 h, compared to a cumulative release rate of only 37% in the absence of GSH. In order to improve the controllability of DB, the group also developed a polymerizable multifunctionalized AB_2_ unit (AB_2_ CTA), thus enabling generation of hyperbranched topologies [134]. The authors carried out the polymerization of OEGMA macromolecular model using AB_2_ CTA to obtain a hyperbranched HPT-4-POEGMA polymer. The polymer also could self-assemble into micelle, and GSH could initiate disulfide bond dissociation and DOX release to improve therapeutic efficacy. The group also developed a disulfide bond-containing macromolecular chain transfer reagent by an [A_2_+B_3_]-based click polymerization, and continued to prepare the HBPs with accurately controllable DB, which also enabled controlled GSH-triggered degradation and drug release [135].

We can also choose to introduce disulfide bonds as crosslinkers into HBPs, which not only make the carrier redox-responsive, but also form additional valence bonds between the chains to improve the carrier stability. Common such crosslinkers are cysteamine, cystamine, and their derivatives. Peng et al. designed a type of galactose-based HBPs with redox-responsive (HRRP) for gene delivery using the disulfide bond-based *N*,*N*′-*bis*(methacryloyl)cystamine (BMAC) as the crosslinkers, and 2-lactobionamidoethyl methacrylate (LAEMA) and AEMA as the monomers (Fig. 7C) [136]. The reaction of this system with high concentrations of GSH was the key to selective delivery of siRNA by HRRP to tumor cells. Due to the presence of disulfide bonds, reduction degradation by GSH promoted the release of the cargo in the cytoplasm. In addition, the use of LAEMA as a glycosyl unit in the system reduced the toxicity of the AEMA cation and increased the biocompatibility of the cationic polymer, thus greatly improving the biosafety of the system application. In HeLa cells, HRRP was shown to achieve 85% silencing efficiency of epidermal growth factor receptor (EGFR), while maintaining 95% survival of normal cells. Thus, HBPs with the characteristics of passive targeting, also known as enhanced permeability and retention (EPR) effect, oxidative regulated drug release, and good biocompatibility is one of the design methods for the development of tumor gene therapy carriers. Further, Jung et al. [137] directly constructed a *CD44*-targeted HA modified hyperbranched Ce6 for tumor PDT therapy using cystamine as the crosslinking agents and Ce6 as the substrates. With the increase of the content of GSH, the crosslinked disulfide bonds were broken and cumulative Ce6 release increased markedly. Experiments revealed that the uptake ratio of Ce6 from this system in HCT116 and U87MG cells was significantly superior to that of the free Ce6, and stronger PDT efficiency was produced. In addition, studies using mouse tumor xenograft model using HCT116 cells also revealed that the system was targeted and enriched more in tumors.

Duan et al. [138] prepared a GSH-sensitive micelle of hyperbranched polyprodrug (DOX-SS-PEG) with a hydrophobic hyperbranched nucleus by coupling 3,3′-dithiodipropanoic acid with the hydroxyl and amino group of DOX. *In vitro* release experiments of DOX showed its significant GSH-responsive release behavior. Cellular experiments also showed that it could be endocytosed by A549 cells and produced significant growth inhibition. They also obtained a GSH-responsive amphiphilic micelle prodrug (DOX-disulfide-based diacrylate (DSDA)-PEG) by coupling DOX and mPEG-NH_2_ by Michael addition in one-pot synthesis using DSDA as the crosslinking agents, which also showed a good application prospect [139].

In addition, we can also use the chemical structure of the drug itself to bind it to the monomer containing a disulfide bond, and further obtain the redox-responsive HBPs with conjugated drugs directly through polymerization. Compared with direct drug encapsulation, this approach has a relatively simple payload, improves DLE by greatly reducing the use of carriers, and provides better control of drug release. For instance, Li and Liu obtained a DOX-coupled polymeric monomer containing a disulfide bond (MA-SS-DOX) through the connection of the amide bond, and further synthesized a redox-sensitive HBP unimolecular micelle precursor (HBPP-DOX) by polymerization in the presence of PEGMA (Fig. 7D) [140]. The size of this unimolecular micelle is about 122 nm with good EPR effect. And on account of the existing disulfide bonds, it has good GSH-triggered drug release properties. At low levels of GSH (10 μM), the DOX release reached equilibrium (2.4%) in 12 h. In the conditions simulating tumor microenvironment, the release could reach 60.9% in 83.5 h. This suggested that the drug was preferred to be released in reducing conditions, such as inside the tumor. This micelle precursor also had a tumor-selective inhibitory effect, and the survival rate of HepG2 human liver cancer cells was as low as 40% after 48 h of treatment, while normal L02 human liver cells were still found to survive more than 90%. In addition, the HBP precursor was simple to synthesize and has a satisfactory DLC, making it a hopeful candidate as drug delivery systems for future tumor chemotherapy. To further promote the degradability of such unimolecular micelles, the group further used the polymeric molecule containing bromine (Br) and disulfide bond (MA-SS-Br) as the inimer to obtain a DOX prodrug possessing a redox-sensitive backbone [141]. At low GSH levels, DOX release was extremely slow (<6.5%) regardless of pH conditions, suggesting that it could remain stable in a normal physiological environment. However, in artificially simulated intracellular conditions of tumor cells, the system realized a cumulative release of 93.20% over 96 h with sustained release capability, which was much greater than that in HBPP-DOX.

In other work, a CPT prodrug (HBPP-CPT) was developed using a similar approach to achieve specific release of CPT in the tumor microenvironment [142]. Contrastingly, Sun et al. [143] prepared a class of HBPs (hPCBE) for synergistic chemotherapy/photodynamic treatment through the copolymerization of redox-sensitive prodrugs of CPT (CPTMA) and photosensitizers. hPCBE was also able to form a stable unimolecular micelle that could be easily endocytosed into MCF-7 cells, which responded to intracellular GSH levels to release CPT, and then induce cell apoptosis. In addition, photosensitizers could also effectively produce SO under the effect of radiation, thus expanding the anti-tumor effect.

Recently, directly employing disulfide bonds to couple anti-tumor drugs into HBPs to construct redox-responsive prodrugs has also attracted much attention. Zhou et al. coupled DOX to natural hyperbranched polysaccharide glycogen (Gly) by the disulfide bond and formed polypyrrole NPs (ppy NPs) with photothermal properties *in situ* inside it (Fig. 7E) [144]. Furthermore, coated with the self-assembled phospholipid layers, a redox-responsive drug delivery carrier (Gly-SS-DOX@ppy@Lipid-arginine-glycine-aspartic acid (RGD)) was constructed for chemotherapy and photothermal synergistic therapy of hepatocellular carcinoma. Gly endowed the system with tumor-targeting properties and expanded the endocytosis efficiency of the cells. The intracellular high concentration of GSH could induce disulfide bond cleavage and drug release. The released DOX could induce cell apoptosis, while ppy NPs could convert near-infrared light into heat energy to further expand the therapeutic effect. Animal experiments with HepG2 tumor models shown that the system could produce significant tumor inhibition without dramatic toxicity to normal organs.

Among the various types of redox-responsive HBPs, cationic hyperbranched polyamide amine (HPAA) composed of disulfide bonds have drawn a great of attention, especially for gene delivery [145]. For example, Li et al. [146] obtained a *N,N*-cystaminebisacrylamide crosslinked redox-responsive hyperbranched HPAA targeted drug carrier through Michael addition polymerization of 1-(2-aminoethyl)piperazine, followed with the surface modification of FA. It could effectively deliver matrix metalloproteinase-9 (*MMP-9*) siRNA and degrade under intracellular reductive conditions, thus exerting effective transfection, leading to the decreased *MMP-9* expression and apoptosis of MCF-7 cells. In addition, RGD [[147], [148], [149]] and targeted aptamer [150], etc., were also used to modify the carrier to further improve the ability of targeted delivery of gene drugs and anti-tumor effect. This type of carriers also shown good application prospects in immunological drug delivery. For instance, Lv et al. [151] made a type of redox-responsive cationic polymer dots (PDs) by partially carbonizing of hyperbranched PAA-PEI_600_ polymer formed from the terminal modification of HPAA with PEI, which were then combined with an antigen protein ovalbumin (OVA) to elicit particular immunity response of OVA in the organism. The study found that the size of the tumors inoculated with OVA alone increased rapidly; in contrast, the PDs/OVA nanoparticles had a marked anti-tumor effect, suggesting that the modified HPAA may be applied in the subcutaneous vaccine delivery system for immunotherapy. A bacteria-like tumor vaccine was also developed to efficiently induce immune response to resist tumor cells, which could stepwise initiate a cascade reaction for precisely deliver of OVA [152].

Yuan et al. [153] used heptafluorobutyric anhydride to fluoridate HPAA containing disulfide bonds, and the obtained polymer HPAA-F7 possessed higher cell surface affinity and cell internalization ability. Flow cytometry analysis showed that it could effectively internalize and delivery the loaded antigen protein into the cytoplasm, thus inducing cell immunity. In addition, this system could also be used for single delivery or co-delivery of chemotherapeutic agents. For instance, Tang et al. constructed a drug co-delivery system (HPAA-MTX/*MMP-9*) by employing HPAA containing disulfide bonds to which MTX was coupled by amide condensation, and further encapsulated the *MMP-9* short hairpin RNA (shRNA) plasmid via electrostatic interaction (Fig. 7F) [154]. And the disulfide bonds would break in response to GSH, which could induce the dissociation of this delivery system, releasing *MMP-9* shRNA plasmid and MTX, thus retarding the cell proliferation and migration of MCF-7 cells. These results showed that it could produce significant synergistic anti-tumor effects. On the basis of HPAA-MTX, Liu et al. [155] further coupled transferrin (Tf) with HNE-1 cells targeting and also loaded with *MMP-9*, and the resulting drug delivery system had significant ability to target HNE-1 cells for drug delivery and gene transfection, which produced stronger synergistic anti-tumor ability. In addition, HPAA has received great attention in the delivery of PTX [[156], [157]], DOX [158], etc.. By further structural modification and property optimization of HPAA, disulfide bond-based HBPs of HPAA will have a promising application in tumor therapy.

## 4 Thermo-responsive HBPs

According to the characteristics of tumor microenvironment, fast proliferation and strong metabolism of tumor cells will result in raised temperature inside the tumor site. Therefore, the development of HBPs with thermo-sensitive characteristics has potential for controlled drug delivery. The molecular structure of thermo-responsive polymers generally contains both hydrophilic and hydrophobic groups. At a low temperature, the polymer show hydrophilicity because of the hydrogen bonding of hydrophilic groups with water molecules; with a gradual increase of temperature, this bonding will be destroyed, thus the hydrophilic property of the polymer will reduce and switch to hydrophobic [[159], [160], [161]]. We generally refer to this transition temperature as the low critical solution temperature (LCST). Therefore, the control of temperature can be used to regulate the interaction between thermosensitive carriers and drugs, as well as the release of drugs. This drug release triggered by temperature stimulation is not only less invasive to living systems, but also promising to achieve intelligent targeting of tumor therapy through the design of hyperbranched carriers.

A widely investigated thermo-sensitive polymer is poly(*N*-isopropyl acrylamide) (PNIPAM) whose LCST is closed to human body temperature at 30–35 °C [[162], [163], [164]]. For instance, Liu et al. [165] synthesized a long-chain PNIPAM backbone (LCHBPNIAPM) and studied its reversible thermo-responsive self-assembly behavior in aqueous solution. The average diameter (DZ) of the self-assembled LCHBPNIAPM changed from 264 to 637 nm as the temperature rose from 20 to 65 °C, and when the solution cooled to 20 °C, the DZ value returned to 258 nm. During the process, its morphology also underwent a cycle transformation from multi-component vesicles to micelles. The cumulative release of DOX at 20 °C is much lower than that at 37 °C, which were 20% and 50% respectively. This proved the feasibility of long chain thermo-sensitive HBPs as drug carriers.

Other kinds of thermo-sensitive HBPs have also received much attention from researchers. For example, Zhang et al. [166] constructed a type of thermosensitive dendronized poly(acylhydrazone) HBPs with various branched structures through the conventional [A_2_+B_3_] polycondensation with ethoxyl-terminated and dialdehyde modified dendritic OEG as the A_2_ monomers and tri(acylhydrazine) amine as the B_3_ monomers. Because of the existing OEG, the HBPs exhibited excellent temperature sensitivity. And on account of the existing surface acylhydrazone bonds, the HBPs could form hydrogels through crosslinking of dialdehyde-terminated PEG, while the temperature responsiveness still retaining. Therefore, it had a good application prospect in controlled drug release and other fields. Sentoukas et al. [167] also prepared a type of star-shaped copolymers with cross-linked poly(*N*-(2-hydroxypropyl)methacrylate) HBPs as core and thermo-sensitive POEGMA_500_ as arms via “core-first” strategy. When the temperature rose, the polymer size gradually reduced due to the contraction of POEGMA_500_, and as temperature continues to increase, aggregation occurred between the particles due to the enhancement of hydrophobicity. While Hayes and Becer [168] designed a hyperbranched poly(2-oxazoline) adopting bifunctional 2-oxazoline based crosslinker (BisOx) and 2-ethyl-2-oxazoline (EtOx) as polymeric monomers. Through adjusting the reaction parameters, its LCST can be adjusted to 44–70 °C, which lay the foundation for subsequent biomedical applications of such thermo-sensitive HBPs.

In addition to the polymerization of typical thermo-sensitive monomers to obtain thermo-responsive HBPs, we can choose HBPs without thermo-sensitive properties to endow them with thermo-responsiveness by artificially introducing the groups similar to the side-chain structure of typical thermo-responsive polymers. For example, Sideratou et al. [169] designed a battery of hyperbranched PEI derivatives (TPEI), with various degrees of functionalization through two-step reaction of hyperbranched PEI with isobutyric acid and isobutyryl chloride. It was found that the LCST of TPEI usually rise as the extent of substitution decreased. The authors carried out experiments with TPEI at an LCST of 38 °C, and found that they were almost non-toxic to MCF-7 cells at 37 °C (below the LCST), and could encapsulate drugs such as DOX through hydrophobicity and showed similar cytotoxicity to free DOX. In contrast, at 40 °C (above LCST), the degree of internalization of DOX from TPEI was increased compared to free DOX, while the cytotoxicity to tumor cells was significantly increased. Therefore, the effects of thermo-sensitive carriers on drug potency due to the change of temperature can be exploited to reduce the toxicity of nano-delivered drugs during the circulation process and achieve precise therapy at the tumor site. Similarly, Ohta et al. [170] obtained a HBPA containing numerous benzoic acid triethylene glycol (TEG) ester moieties by polycondensation of TEG ester. This polymer is also thermo-sensitive with an LCST of 35–37 °C that around body temperature. Moreover, HBPA had a branched structure with internal cavities, which showed a higher capacity for the encapsulation of hydrophobic drugs, thus facilitating *in vivo* drug delivery.

However, due to the complex temperature differences *in vivo* and the contradiction between carrier stability and hydrophilicity/hydrophobicity, direct anti-tumor drug delivery using thermo-sensitive HBPs has not yet been reported. Reports have mainly focused on the preparation and phase transition behavior of novel thermo-sensitive HBPs or the construction of multi-responsive HBPs by combining with other stimulus-responsive units. It is believed that, with further research, temperature as a stimulus will play an increasingly crucial part in HBP-based drug delivery system.

## 5 Multi-responsive HBPs

With the progressive research on stimuli-responsive polymers, researchers have found that both temporal and spatial regulation of release is required to further enhance drug efficacy and decrease toxicity, thus improving release behavior and anti-tumor activity of drugs. Therefore, the selection of dual/multiple stimulus-responsive drug delivery systems, included pH/redox, pH/thermo, pH/redox/light, etc., has a greater application potential for ensuring drug delivery to the target site, avoiding drug leakage in advance, and improving drug release performance [171]. Thanks to the multi-branched and multi-modified terminal structures of HBPs and their internal cavity structures, it's possible to synthesis multi-responsive HBPs based drug delivery systems.

### 5.1 pH/redox dual-responsive HBPs

pH- and redox-responsive systems are two of the most appealing classes of sensitive drug delivery vehicles, as they both take advantage of stimuli that are naturally present in the tumor microenvironment that are easy to modulate, and are therefore of increasing interest in tumor therapy. The combination of the two can better avoid the premature drug release outside of tumor. Therefore, the construction of such dual-responsive HBPs will be important in enhancing drug delivery, improving tumor cellular uptake, promoting cytoplasmic or nuclear internalization, and faster drug release.

Among them, the simultaneous introduction of pH-sensitive and redox-responsive chemical bonds into HBPs is one of the common strategies to prepare pH/redox dual-responsiveness HBPs. For example, Zhang et al. [172] designed a class of pH/redox dual-responsive HBPs based nanomicelles (NMs) by polymerization of PEG diglycidyl ether (PEGDE) and cystamine. The micelle size was about 106–120 nm, which was electronegative at physiological pH due to the existence of carboxyl groups on its surface. In contrast, at pH value of 5.0–6.5, hydrolysis of the amide bonds on its surface would make it electropositive, which was conducive to cell endocytosis. The disulfide bond in cystamine could be broken under the influence of high concentration of GSH after endocytosis, which triggered micellar dissociation and drug release. The micelles had no obvious cytotoxicity, while the loading rate of DOX could be as high as 15.38% (*m*/*m*), and the cumulative *in vitro* release was much higher in the presence of GSH than in its absence. Experiments using HeLa cells as a model also suggested that the DOX-loaded micelles exhibited more effective value-added inhibition and sustained drug release, with better clinical potential.

**Fig. 8:**
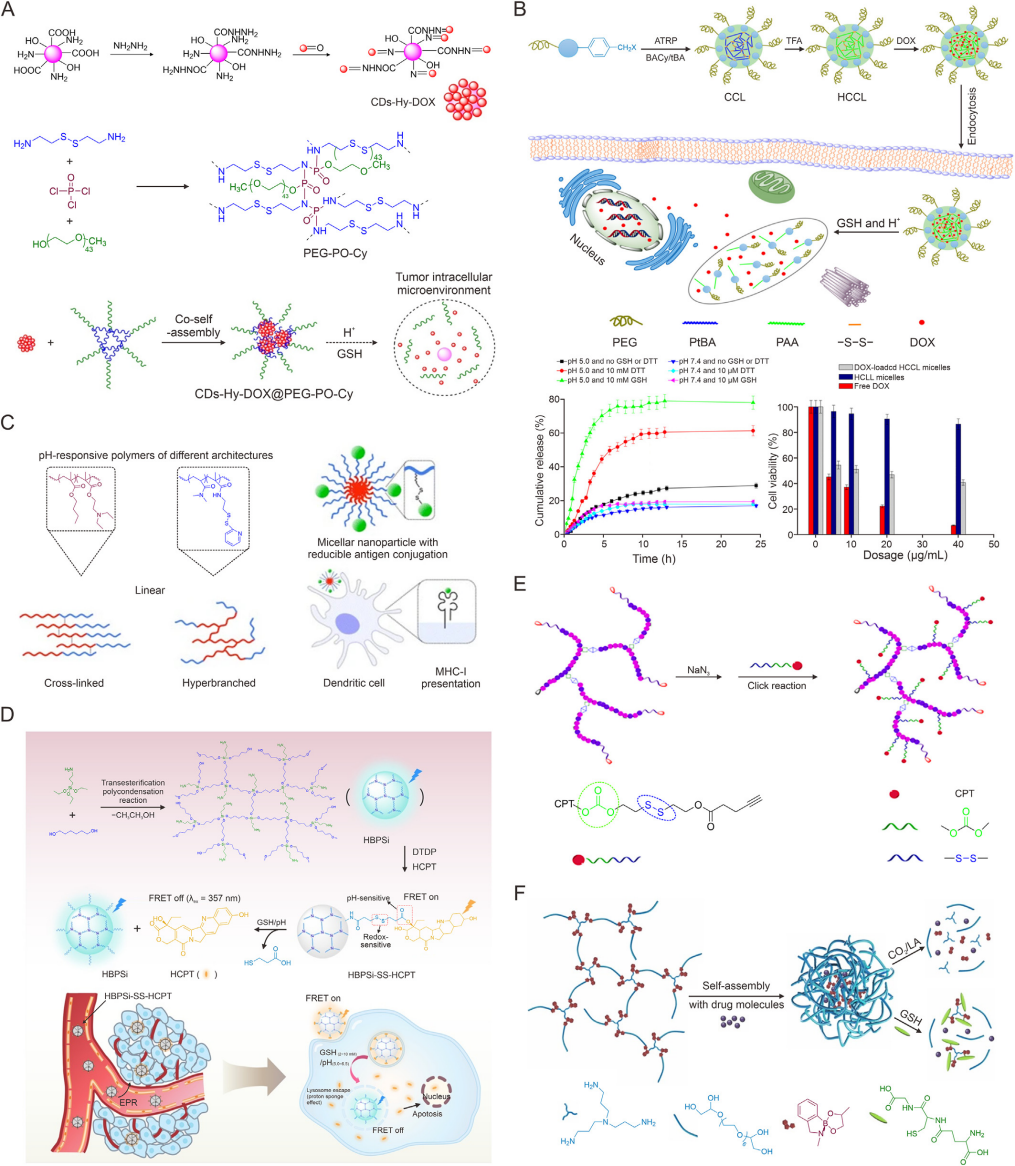
pH/redox dual-responsive hyperbranched polymers (HBPs). (A) Schematic illustration of the pH/redox sensitive HBP doped with carbon dots (CDs) for tumor diagnosis and treatment [173]. (B) Schematic illustration of the core crosslinked hyperbranched micelles (HCCL) with pH/redox responsive for controlled delivery of doxorubicin (DOX) in tumor therapy [175]. (C) Schematic illustration of the pH-responsive nanoparticles for antigen delivery [177]. (D) Schematic illustration of hyperbranched polysiloxane (HBPSi)-based prodrug (HBPSi-SS-10-hydroxycamptothecin (HCPT)) for HCPT delivery [179]. (E) Schematic illustration of the preparation of hyperbranched *h*-P((glycidyl methacrylate (GMA)-camptothecin (CPT))-*st*-oligo(ethylene glycol) methacrylate (OEGMA))-*b*-POEGMA prodrug through click reaction [180]. (F) Schematic illustration of iminoboronate (IB) ester linked micelles with triple stimulus responsive [184]. Hy: hydrazine; PEG: polyethylene glycol; PO: phosphoester; Cy: cystamine; GSH: glutathione; ATRP: atom transfer radical polymerization; BACy: *N*,*N*′-bis(acryloyl)cystamine; tBA: *tert*-butyl acrylate; CCL: core cross-linked micelle; TFA: trifluoroacetic acid; PtBA: poly(*tert*-butyl acrylate); PAA: polyacrylic acid; DTT: dithiothreitol; DTDP: 3,3′-dithiodipropionic; FRET: Förster resonance energy transfer; EPR: enhanced permeability and retention effect; LA: lactic acid. Reprinted from Refs. [173,175,177,179,180,184] with permission.

Li et al. further introduced carbon dots (CDs) with fluorescent effects into a dual-responsive HBP delivery system to create an integrated system for visual treatment of tumors (Fig. 8A) [173]. They synthesized a type of pH/redox triggered HBPs (PEG-PO-Cy) with acid-labeled phosphamide (P–N) and phosphate (P–O–C) groups as well as redox-responsive disulfide bonds, and an acid-labile hydrazine (Hy) connected CD-drug conjugate (CDs-Hy-DOX), and further obtained this diagnostic and therapeutic integrated system by the self-assembly of the two. Since the present of Hy inside CDs-Hy-DOX, as well as the acid hydrolysis of P–N and P–O–C groups and reduction of the disulfides, it has a sharp acid/redox response. It shown that this system was able to slowly release 68.98% of DOX with a premature leakage of 7.58% within three days of treatment in a simulated tumor microenvironment, which could be promote by lowering pH or increasing GSH concentration. In addition, the system showed weak fluorescence, but when DOX was released, the fluorescence of the remaining CDs-Hy would be restored, thus it was expected to be applied to imaging of tumors. Using HepG2 cells as a model, the system was found to be endocytosed into cells. Released DOX and CDs-Hy were mainly located in the nucleus, thus producing significant anti-tumor effects. In contrast, Cheng et al. [174] prepared an amphiphilic poly(amido amine) HBPs using *N*,*N*-cystamine bis(acrylamide) containing disulfide bonds as a crosslinking agent. The system was also pH/redox dual-responsive, capable of accelerating DOX release, promoting cellular uptake and amplifying anti-tumor effects in acidic and simulated tumor microenvironments containing high concentrations of GSH.

Nuclear crosslinked pH/redox dual-responsive HBPs are favored due to their stable morphology and narrow diameter distribution, which can more effectively resolve the contradiction between carrier stability in the circulation and drug release in tumor cells. For example, Tian et al. constructed a redox-responsive HBP with *N,N′*-bis(acryloyl)cystamine (BACy) cross-linked core (HCCL) (Fig. 8B) [175]. The polymer was further endowed with a pH-response by hydrolyzing the *tert*-butyl acrylate (tBA) unit to acrylic acid (AA). DOX could be loaded by electrostatic interaction with 18.4% DLC and 46.0% DLE, respectively. And cumulative releases of DOX within 24 h were 28.8% and 16.8% at acidic and normal pH, respectively, showing good pH-dependent behavior. In the medium with 10 mM GSH and pH 5.0, accumulated release increased to 77.8%, which also indicated its redox-responsiveness. *In vitro* cellular experiments suggested that this polymer had good cytocompatibility and effective cellular uptake, making it a potential carrier for targeted drug delivery. Moreover, Liu et al. [176] constructed a pH/redox-responsive hyperbranched polymeric carrier precursor (DHBP) for the co-delivery of MTX and chlorambucil (Cb) through amidation reaction. Upon further PEG modification, the resulting amphiphilic polymer, DHBP-g-PEG, could self-assemble into stable micelle, which would dissociate under the effect of high levels of GSH and lower pH, thus facilitating MTX and Cb release. And the micelle could also be actually endocytosed by PC-3 cells, thus exerting an effective synergistic anti-tumor effect.

The construction of dual-responsive prodrugs by direct coupling of anti-tumor drugs via responsive chemical bonds into HBP carriers has also received much attention. For example, Wilson et al. designed a HBP by RAFT of butyl methacrylate (BMA) and 2-(*N*,*N*-diethylamino)ethyl methacrylate (DEAEMA), and grafted it with a pyridyl disulfide group modified *N,N*-dimethylacrylamide (DMA) segment (Fig. 8C) [177]. On account of existing amino groups, this polymer exhibited pH-responsive behavior, and the micelle formed by its assembly would gradually dissociate at lower pH and the particle size becomes smaller. Further, thiol-bearing antigens were conjugated to the pyridyl disulfide groups through disulfide bonds, which allowed them to deliver antigens in reducing environments, and cellular experiments also showed that they effectively increased antigen delivery. In another work, Qiu et al. [178] prepared a novel polymerizable CPT-conjugated compound by using β-thiopropionate as the linker, and finally obtain a type of CPT-conjugated pH/redox dual-responsive hyperbranched unimolecular micelles followed by the introduction of a compound containing disulfide bonds. At pH 5.0, the CPT release due to β-thiopropionate fracture was significantly accelerated, e.g., the cumulative release was 58% after 96 h, whereas it was less than 10% at normal pH. Additionally, if 10 mM of GSH was added at the same time, it also would induce the breakage of disulfide bonds within the backbone, leading to accelerated release and increasing the cumulative release rate to 65%. Cellular experiments also demonstrated that these micelles could be internalized into HepG2 and HeLa cells, releasing intracellular CPT, thereby inhibiting tumor growth.

Zhao et al. also prepared a non-conventional polysiloxane (HBPSi) HBP with intrinsic AIE features via the transesterification polycondensation reaction, and further coupled the antineoplastic 10-hydroxycamptothecin (HCPT) to it via ester and disulfide bonds to construct the dual-responsive prodrug (HBPSi-SS-HCPT) (Fig. 8D) [179]. The prodrug showed favorable pH/GSH-sensitivity and was preferentially taken up by MCF-7 cells because of the charge reversal upon release of HCPT. Animal experiments also showed that HBPSi-SS-HCPT could markedly suppress tumor growth without weight loss. In addition, due to the gradual release of HCPT, the carrier gradually regained its AIE properties, thus allowing the use of luminescence to monitor the release of the drug.

Zheng et al. used click reaction to link CPT through disulfide bond and carbonate group with hyperbranched *h*-P(GMA-*st*-OEGMA)-*b*-POEGMA, and obtained a hyperbranched block copolymer prodrug (HP1-CPT) with pH/redox dual-responsiveness (Fig. 8E) [180]. Owing to the existence of reduction-breakable disulfide bonds and pH-sensitive carbonate groups within the skeleton, both acidic pH and GSH-stimulated conditions effectively facilitated the breakage and release of CPT as compared to single triggering. For example, HP1-CPT showed only 25% drug release after 96 h of placement under single condition of reducing or acidity, while 45% release was achieved in the dual condition, and the release time was markedly shorter than the half-life of CPT, thus suggesting that the prodrug, HP1-CPT, could be applied *in vivo* to achieve a long cycle and preferentially accumulate in tumor tissues, which was expected to improve the efficacy and prolong the duration of action.

The group further prepared a type of all-in-one chain transfer monomer which integrated CPT with redox-sensitive disulfide bond and pH-sensitive carbonate group, and further obtained a dual-responsive hyperbranched prodrug by polymerization with OEGMA, which was expected to promote the development of anti-tumor drug carrier [181]. In further work, Thurecht and co-workers [182] constructed a dual-drug synergistic delivery system by coupling DOX and CPT to a biospecific antibody-targeted modified HBP by pH-responsive hydrazone and redox-responsive disulfide bonds, respectively. The results suggested that the dual-drug delivery system produced a superadditive effect in tumor treatment. Recently, they also designed a dual-sensitive system for the delivery of temozolomide (TMZ) and *O*^6^-alkylguanine DNA alkyltransferase inhibitor dialdehyde *O*^6^-benzylguanine (DABG) to reduce multidrug resistance in glioblastoma treatment [183].

In recent years, the introduction of both pH and redox-responsive moieties into HBPs has also gained prominence. Zhang et al. prepared a sensitive micelle for controlled release of CPT by the introduction of the multi-responsive IB ester linkage into a HBP backbone (Fig. 8F) [184]. Stimulated by acidic or reducing environments such as CO_2_, lactic acid, and GSH, the IB ester linkage would break, resulting in the release of CPT. Therefore, the system was well suited for tumor therapy. Studies have also demonstrated that this system could effectively suppress the tumor cell growth and promote cell apoptosis.

### 5.2 pH/thermo dual-responsive HBPs

pH/thermo dual-responsive nano-delivery systems are also of interest. They have higher sensitivity than pH-responsive polymers or thermo-responsive polymers alone, which are suitable for finer and more targeted tumor microenvironments.

Copolymerization of thermo-sensitive and pH-responsive monomers by various means of polymerization is a common means of the construction of pH/thermo-responsive HBPs. For instance, Jiang et al. [185] successfully constructed a kind of pH/thermo-responsive poly(amino ether ester) HBPs through the reaction of ethylene glycol diacrylate with triethanolamine. The polymer had a strong blue fluorescence, whose intensity would change drastically close to the LCST due to the thermo-induced transition of hydrophilicity and hydrophobicity and exhibit a strong acid-induced fluorescence burst because of the existence of acid-sensitive HBPs. The system could thus be used as a responsive fluorescent probe for the tumor microenvironment, to achieve integrated drug delivery and diagnostics. Rwei et al. [186] obtained a pH/thermo dual-responsive HBP by polymerization of NIPAM and ionizable itaconamic acid (IAM). A remarkable reduction in particle size would occur with increasing temperature or decreasing pH, which was ideally suited for drug loading and delivery.

**Fig. 9:**
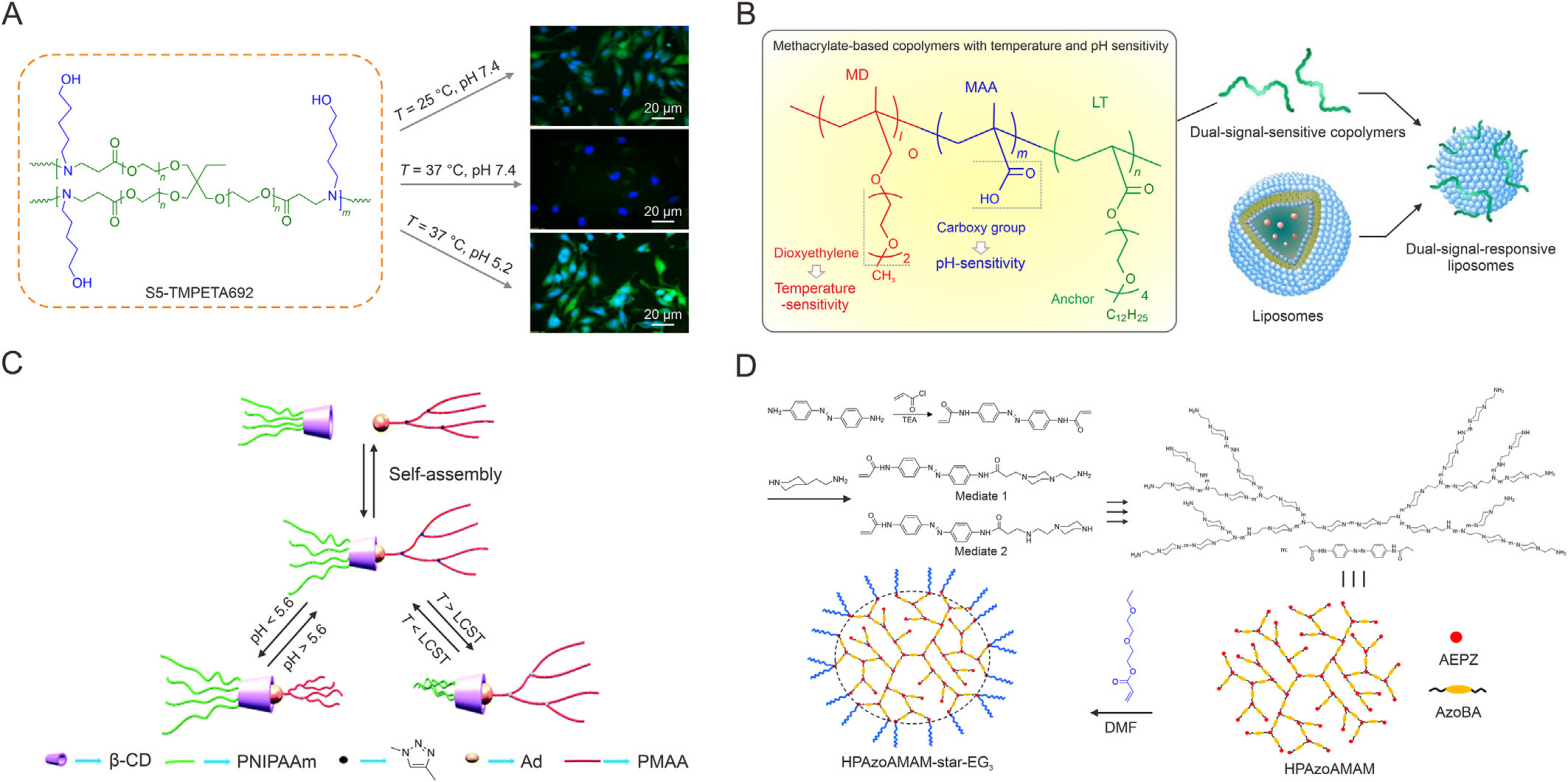
pH/thermo dual-responsive and multi-stimuli-responsive hyperbranched polymers (HBPs). (A) Schematic illustration of the novel kind of coacervate-forming poly(β-amino ester) HBPs (HPAEs) with pH/thermo-responsive for cell binding [187]. (B) Schematic illustration of the construction of pH/thermo dual-responsive liposomes [191]. (C) Schematic illustration of the construction of pH/thermo dual-responsive Janus-like supramolecular HBP [193]. (D) Schematic illustration of the preparation of the azobenzene-containing hyperbranched HBPs (HPAzoAMAM-star-EG_3_) with light-, temperature (*T*)-, and pH-multiple-responsiveness [198]. S5: 5-amino-1-pentanol; TMPETA: trimethylolpropane ethoxylate triacrylate; MD: methoxy diethylene glycol methacrylate; MAA: methacrylic acid; LT: lauroxy tetraethylene glycol methacylate; LCST: low critical solution temperature; β-CD: β-cyclodextrin; PNIPAAm: poly(*N*-isopropyl acrylamide); Ad: adamantane; PMAA: poly(methacrylic acid); AEPZ: *N*-aminoethylpiperazine; AzoBA: *N*,*N*′-(diazene-1,2-diylbis(4,1-phenylene)) diacrylamide; DMF: *N*,*N*′-dimethylformamide. Reprinted from Refs. [187,191,193,198] with permission.

Zhou et al. further prepared a novel type of coacervate-forming poly(β-amino ester) with pH/thermo-responsive (HPAEs) via an [A_2_+B_3_]-type reaction of trimethylolpropane ethoxylate triacrylate (TMPETA) and 5-amino-1-pentanol (S5) (Fig. 9A) [187]. The presence of multiple hydrogen bond pairs in the polymer made it thermo-responsive and the tertiary amines also made it pH-responsive. It had a coacervate nature and was capable of coalescing at higher temperature to encapsulate drug molecules. Studies have shown that it can encapsulate almost all Nile Red model drugs. In addition, at lower pH, due to the increasing positive surface charge, it was better able to bind to HeLa cells and thus been endocytosed by the cells, thereby showing greater potential in molecular transport and release.

Further, Wais et al. [188] first prepared a pH-responsive hyperbranched P(2-(diethylamino)ethyl methacrylate) polymer crosslinked with di(ethylene glycol) dimethacrylate, and then polymerized OEGMA on it to obtain linear thermo-sensitive chain segments, resulting in a series of dual-responsive star-shaped HBPs (SHBs). Using hydrophobic indomethacin as the drug, it was found that SHB showed significantly improved uptake and slower release of indomethacin compared with linear diblock polymers. The authors further incorporated cyclic enone acetal monomers into the polymers, thereby conferring degradability to the main chain polyesters. Such materials combining both main-chain degradability and stimulus-responsive behavior-triggered release with the advantages of simple synthesis and ease of modulation will have immense potential in field of drug delivery.

Selianitis et al. [189] obtained a thermo and pH dual-responsive hyperbranched copolymer (P(OEGMA-*co*-2-(diisopropylamino)ethyl methacrylate (DIPAEMA))) of OEGMA and DIPAEMA. Among them, OEGMA endowed it with good thermo-responsiveness, while the tertiary amino groups of DIPAEMA gave it good pH-responsiveness. Under neutral conditions, it was positively charged and could form stable polyelectrolyte polyplexes with short-linear DNA molecules, thus making it an excellent gene carrier. Also, this copolymer could be used for the delivery of the chemotherapeutic drug indomethacin [190].

Sugimoto et al. designed a copolymer (methoxy diethylene glycol methacrylate-methacrylic acid-lauroxy tetraethylene glycol methacylate (MD-MAA-LT)) of LT, MAA, and MD, and further targeted it to the liposome via egg yolk phosphatidylcholine to obtain a pH/thermo dual-responsive liposomal delivery system (Fig. 9B) [191]. It was shown that cumulative release of model drug pyranine gradually increased with increasing temperature under weakly acidic conditions, whereas there was no such effect under neutral conditions. This delivery system also had an expanded affinity for HeLa cells, which facilitated drug endocytosis. Meanwhile, the higher the temperature, the more it was released in the lysosome, which was an effective strategy that can optimize the performance of liposomal drug delivery systems.

Coupling pH-responsive groups or polymers with thermo-responsive HBPs is also one of the common approaches to prepare pH/thermo dual-responsive HBPs. For instance, Zhu et al. [192] first constructed a thermosensitive hyperbranched polyether (HTHP 1) with double bonds by cationic polymerization of 2-(allyloxy methyl)tetrahydrofuran (AMTHF), tetrahydrofuran (THF), and glycidyl. Further, the authors introduced the pH-responsive carboxyl groups to HTHP 1 via a click reaction of 3-mercaptopropionic acid with the double bonds on HTHP 1, thus prepared a pH/thermo dual-responsive HBP (HTHP 2). With the decreasing of the pH value, the light transmission and zeta potential of the polymer decreased significantly, showing good pH-responsiveness. pH changes would also affect the thermosensitivity, and the LCST of the polymer went from 12.8 to 68.0 °C as the pH was changed from 3.0 to 5.4. More importantly, the polymer was also biocompatible, and the viability of the cells was improved after co-culturing with A549 cells for 24 h. The polymer was also found to be highly biocompatible. After 24 h of co-culture with A549 cells, the cell survival rate was above 90%, which showed good prospects for drug delivery.

Yang et al. constructed a pH/thermo dual-responsive Janus-like HBP by host-guest recognition of adamantane (Ad) group modified hyperbranched poly(methacrylic acid) (PMAA) and β-CD group modified hyperbranched PNIPAM (β-CD-(PNIPAAm)_4_) (Fig. 9C) [193]. It was found that the supramolecular polymer was able to self-assemble to form micelle, still possessing the thermo-responsive behavior of PNIPAM but with higher LCST. The micelle also still exhibited the pH-responsive behavior of PMAA, with a smaller particle size at pH less than 5.4 due to protonation of carboxyl groups. Therefore, this kind of dual-response polymer showed a good application prospect in drug delivery.

### 5.3 Multi-stimuli-responsive HBPs

The introduction of multiple stimulation components into polymers to induce drug release to specific disease sites has been a recent research tendency of drug delivery system. Besides endogenous stimulation, the design of responsive systems has been extended by introducing exogenous stimulus to increase their application potential. Endogenous stimulation can make full use of the tumor microenvironment to accomplish self-controlled release of drugs, while exogenous stimulation can achieve precise temporal, spatial, and dosage control of drug release *in vivo* by external devices [[194], [195], [196]].

Rahemipoor et al. [197] synthesized a type of light-, temperature-, and pH-multiple-responsive HBPs by utilizing one-step polymerization. These polymers were synthesized by polymerization of thermo-responsive 2-(dimethylamino)ethyl methacrylate and photochromic and pH-sensitive spirooxazine acryloyl. As the pH decreases, spirooxazine will absorb H^+^, resulting in a color shift. By adjusting the feed ratio of reactants, the molecular weight and DB of HBPs could be adjusted, which in turn affected their temperature, pH, and photo-responsive behavior. Sun et al. also prepared a type of micelles (LCMs) with light-, pH-, and thermo-multiple-responsiveness from a HBP with azobenzene (HPAzoAMAM-star-EG_3_) (Fig. 9D) [198]. Due to the existence of thermo-sensitive P(2-(2-ethoxyethoxy)ethyl acrylate), it would change from spherical morphology to pancake-like shape while the temperature is below 20 °C. The presence of azobenzene also would cause it elongate into shorter spindles when irradiated by linear polarized light. In addition, the presence of amino groups, which were prone to protonation, would lead to dissociation and smaller particle sizes when the pH is lower to a certain level. The group further built upon this by preparing a HBP with light-, pH-, thermo-, and redox-quadruple-responsiveness (HPAzoBAHB-star-PEG_9_) [199]. In addition to showing good thermo-, pH-, and light-responsiveness, the HBP was also redox-responsive due to the additional introduction of thioether, and was prone to structural dissociation in the presence of H_2_O_2_, transforming from leaf-like lamellar micelle to smaller spherical micelle. This excellent responsiveness makes this HBP have good potential in areas such as drug delivery.

Kohestanian et al. [200] successfully prepared a hyperbranched P(NIPAM-AA) polymer-modified complex GO@Fe_3_O_4_ through the polymerization reaction between AA and NIPAM on the outside of Fe_3_O_4_ modified GO nanosheets. The complex showed low toxicity to HeLa cells, and the cell survival rate remained above 90% even after high concentration treatment. The complex had good temperature- and pH-responsiveness, with faster DOX release at lower pH and higher temperature, and good magnetic properties due to the presence of magnetic GO@Fe_3_O_4_, which was expected to achieve magnetic field-controlled targeted drug delivery.

## 6 Outlook

Making use of the advantage of the distinctions between tumor cells and normal cells, the preparation of stimuli-responsive HBPs based targeted delivery systems has attracted much attention for tumor treatment. Among them, pH- and redox-responsive HBPs have become stars among HBPs and are highly favored. The related studies are increasing year by year, their synthesis methods are becoming more and more simple and ingenious and their structural design and regulatory mechanisms are becoming more and more delicate and dexterous, which play a crucial part in tumor therapy. And the development of multiple-responsive HBPs with more precise regulation of drug release is also gaining attraction.

It has been shown that the introduction of responsive groups or structures into HBPs could enable them to undergo structural changes in response to environmental stimulus, which gives them great potential in the diagnosis, imaging, and delivery of chemotherapeutics, genes, and immunomodulators to different tumors (Table 2) [49,50,52–61,63–86,90–92,94–97,99–102,112,113,115,116,118–120,122,123,125,129–144,146–155,165–170,172–193,197–200]. Among them, because of their biocompatibility, biodegradability, and simple preparation and modification, PEG, PG, PAA, polysaccharides, polyesters, and their derivatives have attracted a lot of attention in the construction of tumor microenvironment-responsive HBPs-based drug delivery systems. In addition, the design of hyperbranched prodrugs and drug self-assembly systems has also attracted increasing attention.

**Table 2:** Representative tumor microenvironment-responsive hyperbranched polymers (HBPs) reported in the literatures.

Skeleton structure	Basic unit	Carrier	Responsiveness	Cargo	Drug loading mode	Application	Refs.
Polyester	2,2-*Bis*(hydroxymethyl)propionic acid	HBP-PEG-Lys-FA	pH	5-FU	Physical embedding	Cervical carcinoma	[49]
	Poly(ortho ester amido amine)	HPOEAMAM/DNA polyplex	pH	DNA	Electrostatic force	Neuroblastoma	[50]
	BDP	RBP@DOX	pH	DOX/RSV	Physical embedding	Breast cancer/cervical carcinoma	[52]
	Poly(β-thioester)	PPHD-PK	pH	DOX	Physical embedding	Cervical carcinoma	[53]
	Poly (amino ester)	PEG-HBPAE-DOX	pH	DOX	Covalently loading	Cervical carcinoma	[54]
	Polyphosphate	*S*-hbPPE/Ce6	pH	Ce6	Physical embedding	Pancreatic cancer	[56]
	*Bis*-MPA polyester-64-hydroxyl	Poly(ester)-*b*-linear poly(carbonate)	pH	GEM	Physical embedding	Pancreatic cancer	[71]
	Sodium carboxyl-modified polyester	PIC micelle	pH	DOX	Electrostatic force	Drug controlled release	[77]
	Boltorn H40	PEG-H40-MSN	pH	DOX	Physical embedding	Breast cancer	[90]
	IB	HBP(OEG-IB)-HQ	ROS	CPT	Physical embedding	Hepatocellular carcinoma	[113]
	Poly(β-thioether ester)	hPTE-1 and lPTE-1 nanoparticle	ROS	–	–	Biomedical application	[118]
	Trimethylolpropane triglycidyl ether	HBPMT micelle	ROS	PTX/Ce6	Physical embedding	Chemo-PDT	[120]
	Polyphosphate with thioketal	RHPPE	ROS	Ce6/DOX	Physical embedding	Breast cancer	[122]
	Polyphosphate with thioketal	HBPTK-Ce6@CPT	ROS	Ce6/CPT	Physical embedding	Colon cancer	[123]
	Polyphosphate	DOX@ss-hbPPE	GSH	DOX	Physical embedding	Breast cancer	[129]
	PCL	SC-PCL-PEG	GSH	DOX	Physical embedding	Hepatocellular carcinoma	[131]
	Dipropargyl 3,3′-dithiodipropionate/1,1,1-tris((4-azidobutanoyloxy)methyl)ethane	MIP-DOX-HP	GSH	DOX	Physical embedding	Increase drug loading	[132]
Polyacetal	2-Hydroxyethyl-4-formylbenzoate	DOX-HBPA-PEG	pH	DOX	Chemical coupling	Hepatocellular carcinoma	[55]
	Divinyl monomer/PEG	Hyperbranched amphiphilic block copolymer	pH	DOX	Physical embedding	Cervical carcinoma	[57]
	Polyacetal/PEG	HBPAs-hydrazone-PEG	pH	DOX	Physical embed	Drug carriers	[61]
	PEG/PCL	H40-star-PCL-a-PEG	pH	DOX	Physical embed	Cervical carcinoma	[65]
PEG	PEGMA	PEGMA/*p*-chloromethylstyrene micelle	pH	DOX	Prodrug	Hepatocellular carcinoma	[58]
	PEGMA	Poly(PEGMA-*co*-TBMC-*co*-EDGMA-*co*-Cy5MA)	pH	DOX	Chemical coupling	Breast cancer	[68]
	PEG/β-CD	TSNs	pH	DOX	Physical embedding	Cervical cancer	[70]
	PEGDA	Poly(PAA-PEGDA)	pH	–	–	Gene/DNA/RNA delivery	[73]
	mPEG-alkynyl	mPEG-hb-S-S-PTAs	GSH	–	–	Bioimaging/copper delivery	[130]
	OEGMA/PCL	h-P(HSEMA-g-PCL)-b-POEGMA	GSH	DOX	Physical embedding	Breast cancer	[133]
	OEGMA	HPT-4-POEGMA micelle	GSH	DOX	Physical embedding	Lung cancer	[134]
	OEGMA	HPT-P(α-OEGMA)	GSH	–	–	Hepatocellular carcinoma	[135]
	PEG-NH_2_	DOX-SS-PEG	GSH	DOX	Prodrug	Lung cancer	[138]
	PEG-NH_2_	DOX-DSDA-PEG	GSH	DOX	Prodrug	Lung cancer	[139]
	PEGMA	HBPP unimolecular micelle	GSH	DOX	Chemical coupling	Hepatocellular carcinoma	[140]
	PEGMA	MA-SS-Br/MA-SS-DOX/PEGMA	GSH	DOX	Chemical coupling	Hepatocellular carcinoma	[141]
	PEGMA	HBPP-CPT unimolecular micelle	GSH	CPT	Chemical coupling	Hepatocellular carcinoma	[142]
	OEGMA	hPCBE	GSH	CPT	Prodrug	Breast cancer	[143]
	POEGMA	PHPMA-b-POEGMA	Thermo	–	–	–	[167]
	PEGDE/cystamine	CPD/CPM/CPS	pH/GSH	DOX	Physical embedding	Cervical carcinoma	[172]
	PEG	CDs-Hy-DOX@PEG-PO-Cy	pH/GSH	DOX	Prodrug	Hepatocellular carcinoma/bioimaging	[173]
	OEGMA/methacryloyloxy-3-thiohexanoyl-CPT	CPT-conjugated HBPs	pH/GSH	CPT	Prodrug	Hepatocellular carcinoma/cervical carcinoma	[178]
	OEGMA	HP1-CPT	pH/GSH	CPT	Prodrug	Cervical carcinoma	[180]
	OEGMA	DRHPP2	pH/GSH	CPT	Prodrug	Cervical carcinoma	[181]
	OEGMA	HBP/CPT/DOX	pH/GSH	DOX/CPT	Chemical Coupling	Breast cancer	[182]
	PEG	HBP-TMZ-DABG	pH/GSH	TMZ/DABG	Physical embedding	Glioblastoma	[183]
	α,ω-di(1,2-diol)s oligo(ethylene glycol)/FPBA	HBP(OEG-IB)	pH/GSH	CPT	Physical embedding	Cervical carcinoma	[184]
	OEGMA/DIPAEMA	P(OEGMA-*co*-DIPAEMA)	pH/Thermo	Gene/indomethacin	Physical embedding	Genetic/drug delivery	[189,190]
Polyacylhydrazone	HPAH/PEG	PEG-HPAH	pH	PTX	Physical embedding	Squamous cell carcinoma of the tongue	[59]
	Tri(acylhydrazine) amine	Poly(acylhydrazone) HBPs	Thermo	–	–	–	[166]
PEI	PEI/PEG	MPEG-PEI-PBLL	pH	DOX	Physical embedding	Lung cancer	[60]
	PEI/Fe_3_O_4_	HPBA-Fe_3_O_4_	pH	DOX	PBA ester bond	Glioblastoma	[97]
	PEI/MWCNTs	oxCNTs@GPEI	pH	DOX	Physical embedding	Prostate carcinoma	[99]
	PEI/isobutyric acid/isobutyryl chloride	TPEI	Thermo	DOX	Physical embedding	Breast cancer	[169]
PG	PG/PBA	HBPO/OEI600-PBA	pH	DOX/p53 gene	Physical embedding	Cervical carcinoma	[63]
	PG/PBA	HBPO(OEI600-PBA)10	pH	Beclin1/siRNA/DOX	Physical embedding	Cervical carcinoma	[64]
	PG/CNCs	CNCs-HPG-HEBA-EPI	pH	EPI	Chemical coupling	Hepatocellular carcinoma	[84]
	PG/MSN	HPG-MSNs	pH	RhB	Physical embedding	adenocarcinoma	[91]
	PG/Fe_3_O_4_	Fe_3_O_4_@PG(BX)CMC-PEG@DOX	pH	DOX	Physical embedding	Cervical carcinoma/MRI	[94]
	PG/GO	rGO-PCH-p-Hyd-g-HPG	pH	CUR/DOX	π−π stacking/covalent bonding	Breast cancer	[[100], [101], [102]]
	PG	HPG-2S-SN38 micelles	ROS	CA	Physical embedding	Breast cancer	[119]
	AMTHF/THF/glycidol	HTHP 2	pH/thermo	–	–	Durg delivery	[192]
Polysaccharide	β-CD	β-CD-HPG-EBA-HH	pH	EPI	Chemical Coupling	Hepatocellular carcinoma	[66]
	Dextran/PEI	Polyelectrolyte complex (HOC)	pH	CpG ODNs	Electrostatic force	Hepatocellular carcinoma	[85]
	β-CD/Fc	β-CD/PEG-Fc	ROS	DOX	Physical embedding	Hepatocellular carcinoma	[115]
	β-CD/Fc	SHPVs	ROS	CDDP/DOX	Physical embedding	Hepatocellular carcinoma/lung cancer	[116]
	Galactose	HRRP	GSH	siRNA	Physical embedding	Cervical carcinoma	[136]
	Gly	Gly-SS-DOX@ppy@Lipid-RGD	GSH	DOX	Chemical coupling	Hepatocellular carcinoma	[144]
PAA	*N*-(2-hydroxypropyl) methacrylamide	HPMA-DOX conjugate	pH	DOX	Chemical coupling	Breast cancer	[67]
	PDMA	LA-(CD-PDMA)_2_	pH	DOX	Physical embedding	Breast cancer	[72]
	2-propylacrylic acid	PAA-co-DMAEMA	pH	RhB	Physical embedding	Drug delivery	[74]
	PMAA	H-P(MAA-*co*-LMA)	pH	CUR	Hydrophobic interaction	Imaging/drug delivery	[75]
	PCL/PAA/PEG	HCAE-FA	pH	PTX	Physical embedding	Cervical carcinoma	[76]
	hPDPA	hPDPA/PArg/HA	pH	β-lapa	Physical embedding	Cervical carcinoma	[86]
	Polymethyl acrylate	MSN-COOH-Tet-HBP-FA	pH	Tet	Electrostatic force	Cervical carcinoma	[92]
	Hyperbranched Ce6	Ce6tetraHA	GSH	Ce6	Chemical coupling	Colon cancer	[137]
	PNIPAM	LCHBPNIAPM	Thermo	DOX	Physical embedding	Controlled release	[165]
	Polyamide (HBPA)	HPBA with TEG ester	Thermo	–	–	–	[170]
	*Tert*-butyl acrylate	HCCL micelle	pH/GSH	DOX	Physical embedding	Ovarian cancer	[175]
	DEAEMA	Poly(DEAEMA-*co*-BMA)	pH/GSH	Antigen	Chemical coupling	Antigen delivery	[177]
	PNIPAM	Hyperbranch-g-(NIPAAm-*co*-IAM)	pH/thermo	–	–	Drug release	[186]
	P(2-(diethylamino)ethyl methacrylate)	SHBs	pH/thermo	Indomethacin	Physical embedding	Controlled delivery	[188]
	PMAA	MD-MAA-LT	pH/thermo	Pyranine	Physical embedding	Cervical carcinoma	[191]
	PMAA/PNIPAM	Supramolecular polymer micelle	pH/thermo	–	–	–	[193]
	DMAEMA	P(MACDT-*co*-DMAEMA)	pH/thermo/light	–	–	Sensors	[197]
	P(NIPAM-AA)	P(NIPAM-AA)-GO@Fe_3_O_4_ @HBC	pH/thermo/magnetic	DOX	Physical embedding	Cervical carcinoma	[200]
Polyamino acid	Boc-Val-HEA	P(Boc-Val-HEA-*co*-VBBT)	pH	–	–	Durg delivery	[69]
	Poly(l-lysine iso-phthalamide)	HPLP	pH	Calcein	Physical embedding	Cervical carcinoma	[79]
	Poly(l-lysine citramide)	HBPLC-PEG-FA	pH	DOX/MTX	Physical embedding	Breast cancer	[80]
	Poly(l-lysine citramide)	HBPAC-GluQD	pH	DOX/MTX	Physical embedding	Breast cancer	[81]
	Poly(l-lysine citramide)	Fe_3_O_4_-HBPLC-Arg/QD	pH	DOX	Physical embedding	Durg delivery	[82]
PAMAM	PAMAM/chitosan	ZWCs-PAMAM-5-FU complex	pH	5-FU	Electrostatic force	Durg delivery	[78]
	PAMAM/Fe_3_O_4_	Fe_3_O_4_@PAMAM-b-PEG-FA	pH	Quercetin	Physical embed	Breast cancer/MRI	[95,96]
	PAMAM	FA-PAAs	GSH	MMP-9 siRNA	Physical embed	Breast cancer	[[146], [147], [148], [149], [150]]
	PAMAM/PEI	PDs/OVA	GSH	OVA	Physical embed	Vaccine delivery	[151,152]
	Fluoridated PAMAM	HPAA-F7	GSH	Antigen protein	Physical embed	Induce cellular immunity	[153]
	PAMAM	HPAA-MTX/MMP-9	GSH	MTX/MMP-9	Chemical coupling/physical embedding	Breast cancer	[154,155]
	PAMAM	poly(BAC2-AMPD1)-PEG	pH/GSH	DOX	Physical embedding	Hepatocellular carcinoma/breast cancer	[174]
	PAMAM	HPAzoAMAM-star-EG3	pH/thermo/light	–	–	Biomedical application	[198,199]
Polyether	Poly(3-methyl-3-(hydroxymethyl)oxetane)	HPMHO-Amines/HPMHO-carboxys	pH	–	–	Drug delivery	[83]
Poly (amino ester)	Poly (amino ester)/PBA	A2-B3-C3-S2-P2	ROS	Protein drug	Coordination interaction	Hemotherapy/gene editing	[112]
	Ethylene glycol diacrylate	Hyperbranched poly(amino ether ester)s	pH/thermo	–	–	Bioimaging/drug delivery	[185]
	TMPETA	HPAEs	pH/thermo	Nile red	Physical embedding	Cervical carcinoma	[187]
polyorganotelluric	B3-Te monomer	B3-Te	ROS	–	–	Breast cancer/lung cancer	[125]
poly(2-oxazoline)	EtOx	Hyperbranched poly(2-oxazoline)s	Thermo	–	–	–	[168]
Drug-based HBPs	MTX/Cb	DHBP-g-PEG	pH/GSH	MTX/Cb	Prodrug	Prostatic cancer	[176]
Polysiloxane	Polysiloxane	HBPSi-SS-HCPT	pH/GSH	HCPT	Physical embedding	Breast cancer	[179]

–: no data. PEG: polyethylene glycol; Lys: dl-lysine; FA: folic acid; 5-FU: 5-fluorouracil; HPOEAMAM: poly(ortho ester amido amine); BDP: *n*-butyl dipropiolate; RBP: PEGylated hyperbranched polymer; DOX: doxorubicin; RSV: resveratrol; PPHD: hyperbranched poly(β-thioester)s; PK: PEG-and KLAK peptide conjugated; HBPAE: hyperbranched poly(amino ester); S-hbPPE: hyperbranched polyphosphoric ester; Ce6: chlorin e6; MPA: methoxy propionic acid; GEM: gemcitabine; PIC: polyion complex; H40: Boltorn H40; MSN: mesoporous silica nanoparticle; OEG: oligo(ethylene glycol); IB: iminoboronate; HQ: 8-hyroxyquinoline; ROS: reactive oxygen specie; CPT: camptothecin; hPTE-1: hyperbranched poly(β-thioether ester); lPTE-1: hyperbranched poly(β-thioether ester); HBPMT: hyperbranched amphiphilic thioether based polymer; PDT: photodynamic therapy; RHPPE: hyperbranched polyphosphate; HBPTK: hyperbranched polyphosphoester containing thioketal units; ss-hbPPE: disulfide bond connected hyperbranched polyphosphoester; GSH: glutathione; PCL: polycaprolactone; SC: disulfide bonds containing star copolymer; MIP: molecularly imprinted polymer; HP: hyperbranched polymer; HBPA: hyperbranched polyamide; PEGMA: polyethylene glycol methacrylate; TBMC: *N*-(*tert*-butoxycarbonyl)-*N*′-(6-methacrylamidohexanoyl)hydrazine; EDGMA: ethylene glycol dimethacrylate; Cy5MA: cyanine-5 methacrylamide; β-CD: β-cyclodextrin ; PAA: polyacrylic acid; PEGDA: poly(ethylene glycol diacrylate); mPEG: methoxy poly(ethylene glycol); PTAs: polytriazoles; OEGMA: oligo(ethylene glycol) methacrylate; POEGMA: poly(oligo(ethylene glycol) methacrylate); HPT: hyperbranched polymer template; DSDA: disulfide-based diacrylate; PEGMA: polyethylene glycol methacrylate; HBPP: hyperbranched polymer prodrug; hPCBE: hyperbranched poly(prodrug-*co*-photosensitizer) amphiphiles; PHPMA: poly(*N*-(2-hydroxypropyl)methacrylate); PEGDE: polyethylene glycol diglycidyl ether; CPD: dimethyl maleic anhydride modified cystamine-based hyperbranched micelle; CPM: maleic anhydride modified cystamine-based hyperbranched micelle; CPS: succinic anhydride modified cystamine-based hyperbranched micelle; CDs: carbon dots; Hy: hydrazine; PO: phosphoester; Cy: cystamine; DRHPP2: dual-responsive hyperbranched polymeric prodrugs; HBP: hyperbranched polymers; TMZ: temozolomide; DABG: dialdehyde *O*^6^-benzylguanine; FPBA: 4-formylphenylboronic acid; DIPAEMA: 2-(diisopropylamino)ethyl methacrylate; HPAH: hyperbranched polyhydrazide; PBA: phenylboronic acid; PEI: polyethyleneimine; MPEG: methoxy PEG; PBLL: poly(Nε-Cbz-L-lysine); HPBA: hyperbranched phenylboronic acid; MWCNTs: multi-walled carbon nanotubes; oxCNTs: oxidized multi-walled carbon nanotubes; GPEI: guanidinylated hyperbranched polyethyleneimine; TPEI: isobutyl-functionalized PEI; PG: polyglycerol; siRNA: short interfering RNA; CNCs: cellulose nanocrystals; HPG: hyperbranched polyglycerol; HEBA: hydrazidation ethyl bromoacetate; EPI: epirubicin; RhB: rhodamine B; GO: graphene oxide; rGO: reduced graphene oxide; PCH: poly(epichlorohydrin); CUR: curcumin; CA: cinnamaldehyde; AMTHF: 2-(allyloxy methyl)tetrahydrofuran; THF: etrahydrofuran; HTHP 2: hyperbranched polyether; EBA: ethyl bromoacetate; HH: hydrazine hydrate; HOC: hyperbranched dextran (HBB)/PEI/cytosine-phosphate-guanine (CpG); ODNs: oligodeoxynucleotides; Fc: ferrocene; SHPVs: supramolecular hyperbranched polymer vesicles; CDDP: *cis*-platinum; HRRP: galactose-based hyperbranched polymer; ppy: polypyrrole; RGD: Arg-Gly-Asp peptide; HPMA: *N*-(2-Hydroxypropyl)methacrylamide; PDMA: poly(2-(dimethylamino)ethyl methacrylate); LA: lithocholic acid; DMAEMA: 2-(dimethylamino) ethyl methacrylate; PMAA: polymethacrylic acid; MAA: methacrylic acid; LMA: lauryl methacrylate; HCAE: H40-poly(ε-caprolactone)-b-poly(acrylic acid)-b′-methoxy poly(ethylene glycol)/poly(ethylene glycol); PTX: paclitaxel; hPDPA: poly(diisopropylaminoethyl methacrylate); PArg: polyarginine; HA: hyaluronic acid; β-lapa: β-lapachone; Tet: tetrandrine; PNIPAM: poly(*N*-isopropyl acrylamide); LCHBPNIAPM: long-chain hyperbranched poly(*N*-isopropyl acrylamide); DEAEMA: 2-(*N*,*N*-diethylamino)ethyl methacrylate; BMA: butyl methacrylate; NIPPAm: poly(*N*-isopropyl acrylamide); IAM: itaconamic acid; SHBs: star hyperbranched polymers; MD: methoxy diethylene glycol methacrylate; LT: lauroxy tetraethylene glycol methacylate; MACDT: 2-((2-(((dodecylthio)carbonothioyl)thio)-2-methylpropanoyl)oxy)ethyl methacrylate; DMAEMA: 2-(dimethylamino)ethyl methacrylate; P(NIPAM-AA): poly(*N*-isopropyl acrylamide-co-acrylic acid); HBC: hyperbranched copolymer; Boc: tert-butyl carbamate ; Val: l-valine; HEA: acryloyloxyethyl ester; VBBT: *S*-(4-vinyl)benzyl S′-butyltrithiocarbonate; HPLP: poly(l-lysine iso-phthalamide); HBPLC: hyperbranched poly(l-lysine citramide); HBPAC: hyperbranched poly l-arginine citramide; GluQD: glucose-derived quantum dot; QD: quantum dot; PAMAM: polyamido amine; ZWCs: zwitterionic chitosan; PAAs: hyperbranched poly(amido amine)s; MMP-9: matrix metalloproteinase-9; PDs: polymer dots; HPAA: hyperbranched poly(amido amine); BAC: *N*,*N*-cystaminebis(acrylamide); AMPD: 4-(aminomethyl)piperidine; HPAzoAMAM: amino-terminated hyperbranched poly(amidoamine) with azobenzene branches; EG3: 2-(2-ethoxyethoxy)ethyl; HPMHO: hyperbranched poly[3-methyl-3-(hydroxymethyl)oxetane]; TMPETA: trimethylolpropane ethoxylate triacrylate; HPAEs: hyperbranched poly(β-amino esters) ; EtOx: 2-ethyl-2-oxazoline; DHBP: dual drug-based hyperbranched polymer; Cb: chlorambucil; HBPSi: hyperbranched polysiloxanes; HCPT: 10-hydroxycamptothecin.

However, because of the complex physiological environment *in vivo*, the advancement of tumor microenvironment-responsive HBPs as drug delivery carriers *in vivo* is challenging, and there are still many problems to be solved. One is that the stimuli-responsive groups or motifs in the molecular structure are often not sensitive enough to the internal microenvironment, which may result in early release of drugs, increase cytotoxicity, and reduce drug efficacy. Secondly, when the stimulus-responsive HBPs are further functionalized, it may affect properties such as hydrophilicity and size, and thereby affect the *in vivo* anti-tumor properties such as tumor penetration. Thirdly, single-stimulus responsive HBPs may be struggle to meet certain practical needs of tumor treatment, while the synthesis routes for multifunctionalized responsive HBPs are still complex, limiting their further application. Fourthly, although stimuli-responsive HBPs have many advantages in drug delivery, but there is still a long enough journey to go before they can be used in the real clinic.

In response to the above problems, we urgently need to further investigate and develop new HBPs based nano-delivery systems. Firstly, we need to develop simpler and easier methods to optimize the synthesis route of HBPs, improve the stability and reproducibility of the synthesis, realize the green synthesis of HBPs, avoid as much as possible the toxic side effects brought by the raw materials or synthesis process [201,202], and at the same time, further improve structural controllability in response to stimulation factors in time and space. Secondly, because of the diversity of biological stimulus in the tumor microenvironment, we can introduce new stimulus-responsive motifs or structures into responsive HBPs, thus developing more HBPs with potential applications. For instance, the lysosomes of tumor cells contain a variety of biological enzymes at higher concentration than that in healthy cells, such as protease and lipase, so the preparation of biological enzyme-responsive HBPs for drug delivery has gradually attracted attention in recent years [203,204]. Moreover, by introducing structures with exogenous stimuli-responsiveness (such as to ultrasound [205,206] and magnetic fields [207,208]) into HBPs and integrating endogenous and exogenous stimuli, it is possible to facilitate the synchronization of diagnosis and therapy while achieving response release and carrier dissociation. In addition, by further introducing targeting ligands, such as FA [209], Tf [210], peptides [211], antibodies [212], etc., into responsive HBPs, the ability of selective action of HBPs may be further improved, the accumulation of drugs in tumors may be expanded, and the drug release may be better targeted.

## 7 Conclusion

As a result of their morphology, structure, and function, HBPs have the potential to play a major role in drug delivery. Through the introduction of tumor microenvironment-responsive groups or units, the resultant responsive HBPs have attracted much attention for tumor therapy. When reaching the tumor site by either active or passive targeting, this kind of responsive drug delivery system will undergo structural transformation or destruction in response to specific characteristics of the tumor microenvironment, thus releasing the loaded drug on demand. This is conducive to increasing the drug specificity and therapeutic efficacy. Although there are still a number of shortcomings and challenges that must be overcome, the authors believe that with extensive efforts from researchers in a wide range of disciplines, a breakthrough in tumor microenvironment-responsive HBPs will be achieved, and it is expected that the shift from experimental research to the clinical environment will be realized in the coming years.

## CRediT authorship contribution statement

**Yuqiong Guo:** Writing – review & editing, Writing – original draft, Visualization. **Xinni He:** Writing – review & editing, Writing – original draft, Visualization. **Gareth R. Williams:** Writing – review & editing, Supervision. **Yue Zhou:** Writing – review & editing. **Xinying Liao:** Writing – review & editing. **Ziyi Xiao:** Writing – review & editing. **Cuiyun Yu:** Supervision. **Yang Liu:** Writing – review & editing, Supervision, Resources, Funding acquisition, Conceptualization.

## Declaration of competing interest

The authors declare that there are no conflicts of interest.
